# Electromagnetic Quasinormal Modes: A Foundational Framework for Describing Fano Resonances of Extinction Spectra

**DOI:** 10.1002/nap2.70071

**Published:** 2026-04-25

**Authors:** Mikhail Bochkarev, Nikolay Solodovchenko, Kirill Samusev, Mikhail Limonov, Tong Wu, Philippe Lalanne

**Affiliations:** ^1^ School of Physics and Engineering ITMO University Saint‐Petersburg Russia; ^2^ Ioffe Institute Saint‐Petersburg Russia; ^3^ Laboratoire Photonique Numérique et Nanosciences (LP2N) Université de Bordeaux Institut d’Optique Graduate School CNRS Talence France

## Abstract

Fano resonances are interference signatures characterized by an asymmetry parameter q and an intensity parameter σ. Traditionally, the Fano parameters are described phenomenologically through fits, limiting physical insight and practical applicability. Available analytical formulations of the Fano parameters are only applicable for extinction spectra and fail to capture realistic scenarios such as nanoresonators on substrates or arbitrary illumination. Here, we develop an ab initio Maxwellian framework based on quasinormal modes (QNMs) that provides a natural physically transparent interpretation of extinction spectra of arbitrarily shaped objects. We show that each individual QNM response inherently exhibits a Fano profile and derive general explicit analytical expressions for q and σ. We further introduce an approximation that enables rapid evaluation of these quantities and allows ultrafast, yet accurate, reconstruction and optimization of extinction spectra using Fano parameters. Numerical and experimental validations confirm the high accuracy, computational efficiency, and ease of implementation of our approach across diverse electromagnetic systems, including photonic exceptional points and dispersive nanoresonators on substrates under arbitrary illumination. This framework provides a clear physical understanding and precise control of resonant line shapes, paving the way for advanced spectral design and optimization in nanophotonics.

## Graphical abstract

An ab initio electromagnetic framework links quasinormal modes to Fano resonances in extinction spectra. Analytical expressions for the Fano asymmetry and intensity parameters are derived from overlap integrals, enabling rapid spectral reconstruction and physical interpretation. The approach provides a universal computationally efficient tool for analyzing and designing resonant nanophotonic systems.

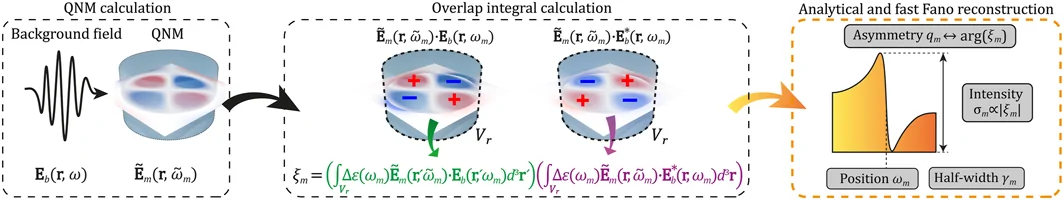

## 1 Introduction

Interference is a universal phenomenon in wave physics. One of its most notable features is the emergence of asymmetric spectral signatures, arising from the interaction of coherent wave processes through different pathways. This feature was first described by Ugo Fano in 1935 [1] and is now referred to as Fano resonance. Fano resonance has been observed in various branches of physics [2, 3, 4, 5], including photonics [6], plasmonics [7], acoustics [8], atomic physics [9], quantum electrodynamics [10], metamaterials [11], lasers [12], and slow light [13]. In optics, recent developments have unveiled striking examples of this phenomenon, such as bound states in the continuum [14, 15], the Kerker effect [16], electromagnetically induced transparency [17], non‐radiating anapoles [18], and cascades of Fano resonances observed in light scattering by dielectric particles [19].

A conventional way to interpret the asymmetric Fano signatures is to consider the interference between a broad resonance (continuum) and a narrow one (localized state labeled by the subscript m), which is described by the Fano profile:

(1) $$\sigma =\sigma_{m}\frac{{\left(q_{m}+\Omega_{m}\right)}^{2}}{\left(\Omega_{m}^{2}+1\right)\left(q_{m}^{2}+1\right)}=\sigma_{m}\left[\;\frac{q_{m}^{2}-1+2q_{m}\Omega_{m}}{\left(\Omega_{m}^{2}+1\right)\left(q_{m}^{2}+1\right)}\right]+\sigma_{m}\frac{1}{\left(q_{m}^{2}+1\right)},$$

where $q_{m}=-\cot\left(\delta_{m}/2\right)$ is the Fano parameter, $\delta_{m}/2$ the phase shift between the narrow and broad resonances, and $\sigma_{m}$ represents the intensity of the narrow resonance; $\Omega_{m}=\left(\omega -\omega_{m}\right)/\gamma_{m}$ denotes the dimensionless frequency, with $\gamma_{m}$ and $\omega_{m}$ representing the resonance damping rate and resonance frequency, respectively. The first expression in Equation (1) is the traditional form of the Fano line shape, whereas the second one separates the line shape (first term) from a constant background (second term).

The parameter $q_{m}$ serves to distinguish an asymmetric Fano from a symmetric Lorentzian profile. The sign and magnitude of $q_{m}$ describe the relative positions of the broad and narrow resonances in the spectrum as well as the nature of their interaction. The Fano profile can manifest either asymmetric or symmetric line shapes, depending on the phase shift $\delta_{m}/2$ between the resonances. When $\delta_{m}/2=0$, the Fano profile simplifies to a Lorentzian shape, $1/\left(\Omega_{m}^{2}+1\right)$, with $q_{m}\to \pm \infty$, indicating constructive interference. Conversely, when $\delta_{m}/2=\pm \;\pi /2$, $q_{m}=$ 0, and the Fano profile adopts a quasi‐Lorentzian form, $-1/\left(\Omega_{m}^{2}+1\right)$, representing destructive interference. Pronounced asymmetry in the spectral line shape emerges when $0<\left|q_{m}\right|\leq 10$. Additionally, a true zero in the spectrum appears when a narrow resonance interacts with a single continuum.

Ugo Fano's seminal articles [1, 2] on interpreting spectra with asymmetric profiles have generated and continue to inspire significant interest across many disciplines. Yet, despite its broad impact, the original framework has important limitations. It is mainly effective only for analyzing isolated sharp spectral features, where the underlying physical mechanisms can be inferred directly from the spectral line shape. Substantial advances in atomic photoionization have since been achieved through complex spectral analysis [20, 21], extending the applicability of Fano‐type interpretations in that domain. We also note an important phenomenological extension of Fano theory for Raman scattering from coupled electron‐phonon excitations, presented in a monograph [22]. In that theory, the spectral dependence of the electronic density of states ρ(E) is explicitly incorporated—an effect neglected in the classical Fano formalism—leading to a modified profile: $\sigma ∼\rho \left(E\right)\;{\left(q+\Omega \right)}^{2}/\left(\Omega^{2}+1\right).$ Here, both the line shape and the asymmetry parameter become energy dependent q(E). Despite these developments, the use of Fano theory within optics remains predominantly phenomenological: it lacks a rigorous electromagnetic basis and depends heavily on fitted system‐specific parameters.

Establishing a theoretical framework that delivers general analytical expressions for the Fano parameters for arbitrary resonator shapes and materials would represent a major advance. We find that quasinormal mode (QNM) theory offers an elegant and comprehensive solution to this challenge. QNM theory, much similar to Fano resonances, spreads across various wave disciplines, including mathematics [23], gravitational waves physics [24], quantum mechanics [21], and electromagnetism [25]. QNMs have complex frequencies (or energies), ${{\tilde{\omega }}_{m}=\omega }_{m}-i\gamma_{m}$, where the imaginary part encodes decay into a continuum. In electromagnetism, this decay can originate from a variety of mechanisms, including material absorption and leakage into different channels such as plane waves in free space, waveguide modes in layered media, or “guided” surface plasmons. This diversity greatly complicates the problem and likely explains why the Fano approach has remained predominantly phenomenological until now. Hereafter, we adopt an ab initio Maxwellian approach utilizing QNM theory [26, 27] to derive an analytical expression for both $q_{m}$ and $\sigma_{m}$ in a general form.

Although the connection between electromagnetic QNMs and the Fano response may seem intuitive, it remains largely underexplored. Previous studies have been limited to a few specific cases. For example, in [28], the density‐matrix formalism of two‐level quantum systems is combined with QNM theory to predict that the near‐field response of a quantum emitter coupled to surface plasmons exhibits Fano line shapes that vary with the spatial coordinate. Similar conclusions have been reached in related theoretical studies on emitter‐plasmon systems using slightly different approaches [29, 30]. Furthermore, it has been demonstrated that the Purcell‐effect spectrum of emitters coupled to spectrally overlapping resonances can also exhibit Fano line shapes [31], a phenomenon well understood within the framework of non‐Hermitian complex mode volumes [32]. Across these studies, the Fano line shape consistently arises in the near‐field due to the interaction between spectrally overlapping resonances with different quality factors.

In this study, we adopt a more fundamental perspective, examining the general case of electromagnetic resonances excited by arbitrary far‐field waves. The approach reveals a deep analogy with quantum mechanics, where Fano line shapes were first identified in the resonance scattering spectra of quasi‐bound states in the continuum [2]. Our main finding is that each individual QNM contributes an intrinsic Fano line shape to the extinction spectrum, with Fano parameters given by closed‐form expressions—without the need for spectral overlap or near‐field backaction. This result demonstrates that QNMs naturally encode the interaction between localized resonances and the radiation continuum, making them a powerful representation of bound states in the continuum. Importantly, the Fano parameters vary from mode to mode, reflecting the distinct coupling of each QNM to available decay channels. Consequently, the overall extinction spectrum emerges as a superposition of multiple Fano resonances, each characterized by its own asymmetry and linewidth.

Besides the fundamental contribution, our approach is highly effective in terms of computational speed and accuracy. It enables direct calculation of $q_{m}$ and $\sigma_{m}$ for an extinction spectrum and, therefore, rapid optimization calculations and spectrum reconstruction with high frequency resolution. The theory is applicable to any reciprocal photonic system, including challenging scenarios, such as dispersive nanoresonators on structured substrates or exceptional points (EPs), for which other precise or phenomenological methods fail to obtain the Fano parameters.

## 2 Quasinormal Mode Theory of Fano Resonances

Electromagnetic QNMs are source‐free solutions of Maxwell equations, $\nabla \times {\tilde{E}}_{m}=i{\tilde{\omega }}_{m}\mu_{0}{\tilde{H}}_{m}$, $\nabla \times {\tilde{H}}_{m}=-i{\tilde{\omega }}_{m}\varepsilon_{0}\varepsilon \left(\tilde{\omega }\right)\;{\tilde{E}}_{m}$, which satisfy the outgoing‐wave condition for |r|→∞ [25]. Hereafter, ${\tilde{E}}_{m}$ and ${\tilde{H}}_{m}$, respectively, denote the normalized electric and magnetic fields of the mth QNM, ${\tilde{\omega }}_{m}=\omega_{m}-i\gamma_{m}$ is its complex eigenfrequency, ε is the relative permittivity (which could be considered as a symmetric tensor), $\varepsilon_{0}$ and $\mu_{0}$ are the permittivity and permeability of vacuum, respectively. The QNM field exponentially decays in time and $\text{Im}\left({\tilde{\omega }}_{m}\right)<0$ with the exp(−iωt) notation. We restrict our further discussion to non‐magnetic materials; however, extending the analysis to magnetic systems is straightforward. Our only assumptions are that the materials are reciprocal and can be described by Drude‐Lorentz permittivity $\varepsilon \left(r,\omega \right)=\varepsilon_{\infty }\left(r\right)+\varepsilon_{DL}\left(r,\omega \right)=\varepsilon_{\infty }\left(r\right)-\varepsilon_{\infty }\left(r\right)\omega_{p}^{2}/\left(\omega_{0}^{2}-\omega^{2}+i\omega \gamma_{0}\right)$, where $\varepsilon_{\infty }\left(r\right)$ can be complex valued.

The resonator is illuminated by a monochromatic wave with a frequency ω. The incident wave is referred to as the background field [26] in the presence of a substrate with an electric field denoted by $E_{b}\left(r,\omega \right)$. The extinction cross‐section in a case of lossless host media, $\varepsilon_{b}\left(r\right)\in R$, is given by

(2) $$\sigma_{\text{ext}}=\frac{1}{2P_{0}}\int_{V_{r}\;}\text{Im}\left\{\omega \varepsilon_{0}\left(\varepsilon_{\infty }-\varepsilon_{b}\right)E_{b}^{*}{\;\cdot \;E}_{s}+iE_{b}^{*}{\;\cdot \;J}_{tot}+\omega \varepsilon_{0}\left(\varepsilon_{\infty }-\varepsilon_{b}\right)E_{b}^{*}\cdot E_{b}\right\}d^{3}r,$$

where $E_{s}$ is the field scattered by the resonator, $J_{tot}=-i\omega P_{tot}=-i\omega {\varepsilon_{0}\varepsilon }_{DL}\left(r,\omega \right)\left(E_{s}+E_{b}\right)$ is the total polarization current, $P_{0}$ is the time‐averaged Poynting vector of the incident wave. The integration is performed over the volume of the resonator $V_{r}$, where $\varepsilon \left(r\right)-\varepsilon_{b}\left(r\right)\;\neq \;0$. We use the auxiliary field QNM expansion [26] to represent the scattered electromagnetic field

(3) $$\left[E_{s}\left(r\right),H_{s}\left(r\right),P_{tot}\left(r\right),J_{tot}\left(r\right)\right]=\sum_{m}\alpha_{m}\left(\omega \right)\;\left[{\tilde{E}}_{m}\left(r\right),{\tilde{H}}_{m}\left(r\right),{\tilde{P}}_{m}\left(r\right),{\tilde{J}}_{m}\left(r\right)\right],$$

where $\alpha_{m}$ is the excitation coefficient of the mth QNM. Expansions with only QNMs are generally not complete, especially when there is a substrate (branch cuts) [25]. To account for this possibility and remain as general as possible, we use a formalism based on a QNM‐regularization with complex mappings implemented with perfectly matched layers (PMLs). The extension of Equation (3) then encompasses a finite set of QNMs that are unaffected by the mapping and an infinite set of numerical eigenmodes often called PML modes. The incorporation of PML modes guarantees completeness over the whole mapped space with an expression for the excitation coefficients directly derived from the divergence theorem [26]

(4) $$\alpha_{m}\left(\omega \right)=-\frac{\omega }{\omega -{\tilde{\omega }}_{m}}\int_{V_{r}\;}\varepsilon_{0}\left(\varepsilon_{\infty }\left(r\right)+\left(\frac{{\tilde{\omega }}_{m}}{\omega }\right)\varepsilon_{DL}\left(r,{\tilde{\omega }}_{m}\right)-\varepsilon_{b}\left(r\right)\right)E_{b}\cdot {\tilde{E}}_{m}d^{3}r.$$

Substitution of Equations (3) and (4) into Equation (2) yields an expansion of the extinction cross‐section as $\sigma_{\text{ext}}=\sum_{m}\sigma_{\text{ext}}^{m}+\sigma_{\text{ext}}^{bg}$, where the background contribution $\sigma_{\text{ext}}^{bg}\equiv \frac{\omega }{2P_{0}}\left(\int_{V_{r}\;}\varepsilon_{0}Im\left(\varepsilon_{\infty }-\varepsilon_{b}\right)E_{b}^{*}\cdot E_{b}d^{3}r\right)$ is independent of the QNMs and produces a uniform extinction offset when $Im\left(\varepsilon_{\infty }\right)\neq 0$. This classical step is then followed by a series of algebraic manipulations applied to $\sigma_{\text{ext}}^{m}$. These manipulations, taken individually, do not pose intrinsic difficulties; it is rather the sequence that is not trivial. We were indeed helped by our intuition to identify a Fano‐like response for every $\sigma_{\text{ext}}^{m}$ when we conducted the manipulations. The full derivation is presented in Supporting Information S1: Section I.

We find that $\sigma_{\text{ext}}^{m}$ can be conveniently expressed as the classical Fano formula [Equation (1)]

(5a) $$\sigma_{\text{ext}}^{m}=\sigma_{m}\left[\;\frac{q_{m}^{2}-1+2q_{m}\Omega_{m}}{\left(\Omega_{m}^{2}+1\right)\left(q_{m}^{2}+1\right)}\right],$$

with closed‐form expressions for the real‐valued Fano parameter $q_{m}$ and the resonance intensity $\sigma_{m}$

(5b) $$q_{m}=-\cot\left(\frac{\arg\left(\xi_{m}\right)}{2}\right),$$

(5c) $$\sigma_{m}=\frac{1}{P_{0}}\frac{\omega^{2}}{2\gamma_{m}}\left|\xi_{m}\right|.$$

The term $\xi_{m}$ is defined as the product of two overlap integrals over the resonator volume $V_{r}$

(6) $$\xi_{m}\equiv \left(\int_{V_{r}\;}\Delta \varepsilon \left(\omega ,r^{\prime }\right){\tilde{E}}_{m}\left(r^{\prime }\right)\cdot E_{b}\left(\omega ,r^{\prime }\right)d^{3}r^{\prime }\right)\left(\int_{V_{r}\;}\Delta \varepsilon \left(\omega ,r\right){\tilde{E}}_{m}\left(r\right)\cdot E_{b}^{*}\left(\omega ,r\right)d^{3}r\right),$$

where the dielectric contrast is $\Delta \varepsilon \left(\omega ,r\right)\equiv \varepsilon_{0}\left(\varepsilon_{\infty }\left(r\right)+\left(\frac{{\tilde{\omega }}_{m}}{\omega }\right)\varepsilon_{DL}\left(r,{\tilde{\omega }}_{m}\right)-\varepsilon_{b}\left(r\right)\right)$ with the dispersive part of permittivity $\varepsilon_{DL}$ taken at the resonant frequency ${\tilde{\omega }}_{m}$.

Importantly, the derivation of Equations ((5a), (5b), (5c)) and (6) is free from approximations, relying only on the assumption of material reciprocity. It imposes no restrictions on the resonator shape, material characteristics, or background substrate. The theory is easily generalized to the case of N‐pole Drude‐Lorentz permittivity [26]. Alternative permittivity models can also be considered [33].

In a final step, we further propose to directly relate Equations (5b,5c) with Equation (1). Due to the potential ω‐dependence of Δε and $E_{b}$ in Equation (6), $q_{m}$ and $\sigma_{m}$ in Equations (5b,5c) are also ω‐dependent, preventing the assignment of a unique Fano factor to each resonance. This issue can be easily overcome by assuming that $q_{m}$ and $\sigma_{m}$ vary more slowly than the Lorentzian factor $1/\left(\Omega_{m}^{2}+1\right)$. We therefore neglect the ω‐dependence of $\xi_{m}$ and calculate it at the real part of the eigenfrequency $\xi_{m}\equiv \xi_{m}\left(\omega_{m}\right)$. This approximation, referred to as the “*resonant frequency approximation*” (RFA) hereafter, together with Equations ((5a), (5b), (5c)) and (6) represents the main theoretical result of the present work.

The RFA approach for calculating $q_{m}$ and $\sigma_{m}$ as well as reconstructing the system response is straightforward to implement, computationally efficient, and physically intuitive, as demonstrated in the following sections. A schematic overview of the algorithm is presented in Figure 1. The only required inputs are the analytical expression of the background field $E_{b}$ and the numerically computed QNM electric field ${\tilde{E}}_{m}$. By substituting these fields into Equation (6) and evaluating $\xi_{m}$ at the resonant frequency $\omega_{m}$, accurate values of $q_{m}$ and $\sigma_{m}$ are directly obtained from Equations (5b) and (5c). Together with the known resonant frequency $\omega_{m}$ and half‐linewidth $\gamma_{m}$, these quantities form a set of four characteristic parameters, ${\omega_{m},\gamma_{m},q}_{m},\left|\xi_{m}\right|$ or $\sigma_{m}$, which hereafter refer to as the *spectral parameters*. Within the Fano formalism [Equation 5(a)], these parameters provide a complete description of the spectral response associated with an individual QNM.

**FIGURE 1:**
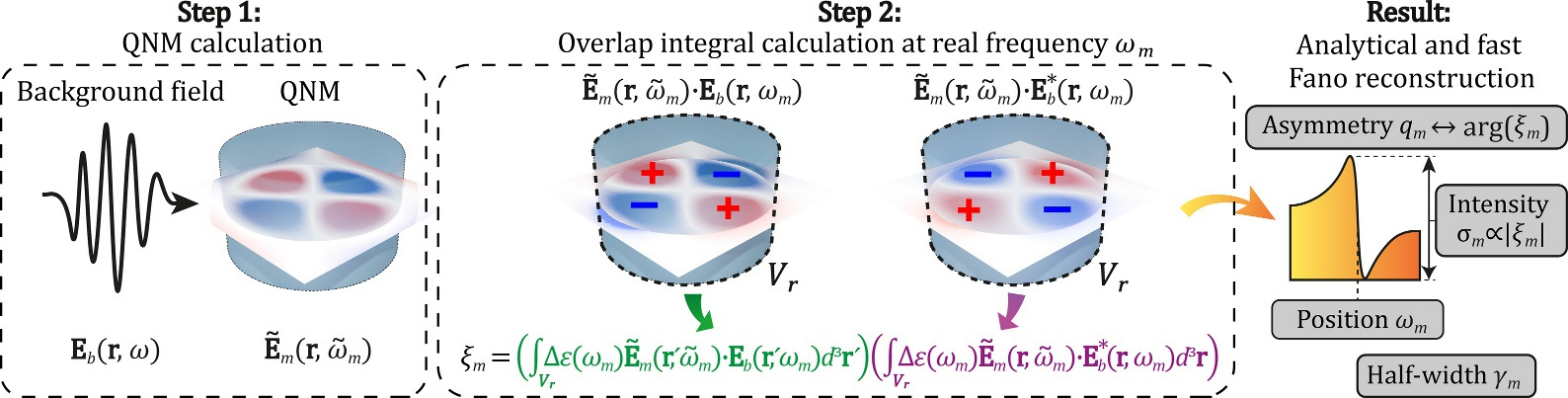
Flowchart of the RFA calculation for spectral parameters with QNM expansions. For an analytically defined background field and each computed QNM, the double overlap integral $\xi_{m}$ is evaluated with Equation (6) at resonant frequency $\omega_{m}$. As a result, with Equations (5b,5c), we obtain the spectral parameters $({\omega_{m},\gamma_{m},q}_{m},\sigma_{m}$ or $\left|\xi_{m}|\right)$, which completely describe an individual QNM‐Fano response. Together with Equation (5a), the parameters can be employed to efficiently reconstruct the total extinction spectrum as a sum of Fano contributions obtained for every QNM.

The choice between $\left|\xi_{m}\right|$ or $\sigma_{m}$ depends on the intended application. For accurate spectral reconstruction, it is preferable to retain the analytical $\omega^{2}-$ dependence in Equation (5c), which ensures higher accuracy for low‐Q QNM responses and for reconstruction of the total spectrum (Supporting Information S1: Section II.1). Conversely, when the emphasis is on intensity behavior, it is convenient to consider frequency‐independent quantities, with $\sigma_{m}\;\propto \;\omega_{m}^{2}\left|\xi_{m}\right|$. The former approach is adopted in Sections 3 and 6, where the algorithm outlined in Figure 1 is employed for rapid spectral reconstruction and technical implementation. The latter is used in Sections 4 and 5, where we focus on elucidating the physical mechanisms governing variations in $q_{m}$ and $\sigma_{m}$.

The parameter $\xi_{m}$ plays a central role in the theory, as it reveals that Fano asymmetry is primarily controlled by two contributions: the QNM field ${\tilde{E}}_{m}$ and the illumination field $E_{b}$. The first contribution can be modified through dissipative coupling between QNMs [34], which may substantially reshape ${\tilde{E}}_{m}$, while leaving $E_{b}$ nearly unchanged. Such coupling‐induced variations in $q_{m}$ are frequently observed in quasi–Bound states in the continuum (quasi‐BIC) [34, 35]. In contrast, the second contribution preserves ${\tilde{E}}_{m}$ but alters the excitation conditions, for example, through changes in the incident angle of $E_{b}$.

Formally, the dependence of the Fano parameter on the incident field $E_{b}$ was first introduced in the Temporal Coupled‐Mode Theory (TCMT) [36]. In most implementations, however, TCMT treats this dependence phenomenologically, through effective coupling coefficients and phase parameters, while neglecting the spatial structure of both $E_{b}$ and ${\tilde{E}}_{m}$. This simplification limits its predictive power for complex electromagnetic environments.

In Sections 4 and 5, we will demonstrate that the Fano parameter can be continuously tuned from 0 to ±∞ through both mechanisms discussed above. These demonstrations are carried out for high‐permittivity particles, where the resulting effects are more pronounced.

The indicated mechanisms are largely geometric. However, Equation (6) also includes a fundamental term that defines the specific type of light‐matter interaction—namely, the permittivity contrast Δε(ω,r). This term can encompass material resonances of various origins, including interband transitions or excitonic responses—all of which manifest themselves in material dispersion. The other physics, such as coupling between QNMs and surface plasmon polaritons or guided modes, can be enabled in the presence of a structured substrate. In Section 6, we examine a dispersive nanoparticle embedded in a layered substrate as an example demonstrating the applicability of the RFA to realistic nanostructures.

Notably, $\xi_{m}$ incorporates two overlap integrals, allowing the incident field $E_{b}$ and its complex conjugate $E_{b}^{*}$ to be interchanged. This property arises from the fact that conjugating the incident field reverses its propagation direction, as $E_{b}^{*}\left(k_{b}\right)=E_{b}\left(-k_{b}\right)$. As a result, $\xi_{m}$ remains unchanged under a reversal of the incident wave direction, that is, $\xi \left(k_{b}\right)=\xi \left(-k_{b}\right)$. It follows directly that $q_{m},\sigma_{m}$ and $\sigma_{\text{ext}}^{m}$ are all likewise invariant. It is well established that, in reciprocal systems, the total extinction remains unchanged regardless of whether the system is illuminated from one side or the opposite side, $\sigma_{\text{ext}}\left(k_{b}\right)=\sigma_{\text{ext}}\left(-k_{b}\right)$ [37, 38]. Here, we extend this principle by demonstrating that the extinction “per resonance” and the associated Fano parameters are also invariant under this directional reversal. An analogous property holds for the transmittance spectra of reciprocal systems [39].

Accordingly, this invariance can be viewed as a consequence of the optical theorem, which relates $\sigma_{\text{ext}}$ and the scattering matrix via $\sigma_{\text{ext}}/A=2\left(1-Re\left\{S_{21}\right\}\right)$, where A is a characteristic normalization area and $S_{21}$ (or t(ω)) is the complex amplitude of transmittance. The latter expression broadens the applicability of our theory to studies of particles placed in waveguides [40] (Supporting Information S1: Section III.4). We also note that for the amplitude reflectance coefficient r(ω), the analytical Fano contribution in the form of Equation (5a) can be straightforwardly obtained using the derivation presented in Supporting Information S1: Section I together with results of [41].

To our knowledge, only two recent studies [35, 42] have proposed closed‐form expressions for the Fano parameter. In [42], an analytical expression for $q_{m}$ was derived for the Mie multipoles of a spherical particle in free space using Bessel and Hankel functions. However, since Mie multipoles do not correspond to resonances with complex frequencies, this approach is both narrower in scope and notably distinct from the present QNM‐based approach. The second study is conceptually closer, as it establishes a connection between Fano parameters to QNMs [35]. Nevertheless, its derivation relies on restrictive symmetry assumptions, which preclude the analysis of many practical resonator configurations, particularly those involving substrates. Furthermore, the resulting expression for $q_{m}$ does not generally satisfy reciprocity constraints, thereby limiting its validity to systems with spatial inversion symmetry and linearly polarized excitation fields $E_{b}$.

The limitations we highlight in earlier works [35, 42] are not meant to detract from their contributions. On the contrary, these studies constitute important early steps toward analytical treatments of Fano resonances, and have played a significant role in shaping subsequent research in the field. Our discussion of these constraints is instead intended to highlight the progress enabled by the present approach, which overcomes all previously noted restrictions and, to the best of our knowledge, offers the only practical framework for the direct and general calculation of all spectral parameters.

## 3 Fast Spectra Reconstruction With Resonant Frequency Approximation

First, we demonstrate the simplicity and high accuracy of the RFA in the reconstruction of optical spectra. We consider a cylindrical nanoparticle with ε=16+0.016i, R=250 nm, H=300 nm, embedded in vacuum and analytically define the background field $E_{b}$ as a linearly polarized plane wave at normal incidence (inset in Figure 2b). Following the flowchart in Figure 1, we compute the QNM eigenfrequencies ${\tilde{\omega }}_{m}$ and eigenvectors ${\tilde{E}}_{m}$ with the freeware MAN [27]. The QNMs are normalized using the PML norm [25]. The term $\xi_{m}$ is then evaluated with Equation (6) for each QNM at its resonant frequency $\omega_{m}$, yielding theoretical values of $q_{m}$ with Equation (5b). For the intensity term $\sigma_{m}$, we calculate $\left|\xi_{m}\right|$ and substitute it into Equation (5c), preserving the analytical $\omega^{2}-$ dependence for better accuracy. As a result, we obtain the spectral parameters (${\omega_{m},\gamma_{m},q}_{m},|\xi_{m}|$) for each QNM. Finally, substituting these parameters into the analytical expression of Equation (5a) produces the RFA response for each QNM. As an “exact” reference data, we use the extinction cross‐section computed using the standard COMSOL frequency‐domain solver. The intercomparison is shown in Figure 2.

**FIGURE 2:**
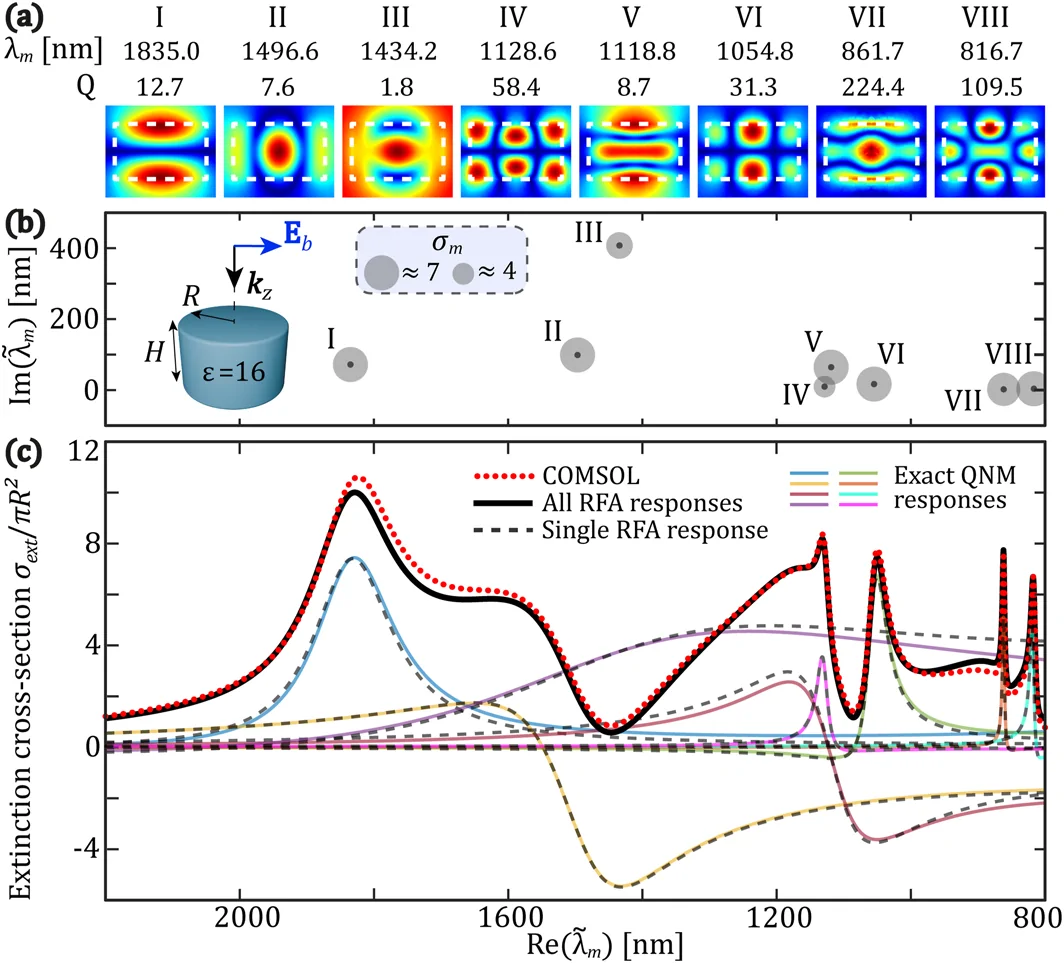
Extinction spectrum reconstruction by RFA for a cylindrical nanoparticle. (a) Distributions of $\left|{\tilde{E}}_{m}\right|$ in a vertical cross‐section for several QNMs of the resonator (from I to VIII) that exhibit the most intense spectral responses in the wavelength range studied. Here, complex resonant wavelength ${\tilde{\lambda }}_{m}=2\pi c/{\tilde{\omega }}_{m}$, with $\lambda_{m}=Re\left\{{\tilde{\lambda }}_{m}\right\}$. (b) Imaginary parts of eigenvalues ${\tilde{\lambda }}_{m}$ for corresponding QNMs. The size of grey circles is proportional to the intensity at resonant frequency $\sigma_{m}$. (c) Comparison between the extinction spectrum calculated with standard COMSOL frequency domain solver (red dotted curve) and the RFA reconstruction (solid black curve). Colored curves correspond to ‘exact’ response calculation for each single QNM with Equation (5a) (all ω−dependencies are preserved); dark dashed curves correspond to RFA responses calculated at real frequency $\omega_{m}$ (only analytical $\omega^{2}-$dependence of $\sigma_{m}$ is preserved). The resonator and illumination conditions are shown in the insert in (b).

Figure 2c shows good agreement between the standard COMSOL frequency‐domain data (red dotted curve) and the RFA reconstruction (black solid curve). The individual QNM contributions to the extinction (colored curves in Figure 2c) are also well reproduced by RFA responses (dark dashed curves), each of which represents individual Fano lines with a unique constant $q_{m}$. Remarkably, a qualitative agreement remains even for QNM III (Figure 1a,b), with its low quality factor of Q= 1.8. As Q increases, the reconstruction accuracy further improves (Supporting Information S1: Section II.1).

A direct comparison of computational times between a standard frequency‐domain approach and the QNM + RFA method is not straightforward, as it depends strongly on the specific problem. Here, we briefly highlight two key differences and the conditions under which the RFA method becomes advantageous. A detailed analysis of computational cost for both methods is provided in Supporting Information S1: Section II.2.

The computational time of standard frequency‐domain solver like COMSOL scales linearly with the number of frequency points, whereas the computational cost of RFA is practically independent of the chosen frequency resolution. In contrast, conventional solvers yield an “exact” frequency‐domain response, whereas perfect reconstruction within RFA is generally unattainable due to the neglected frequency dependence of $\xi_{m}$. RFA is therefore particularly well suited for the rapid generation of highly resolved broadband spectra across different geometries and illumination conditions, especially when exact pointwise agreement is not required. As an illustration, the spectrum shown in Figure 2c, comprising 2001 frequency points, was reconstructed in approximately 3 min using the combined 400‐QNM + RFA approach.

The prime advantage of RFA is its substantial dimensionality reduction of data. The full spectrum, typically represented by high‐dimensional data (e.g., 2001 × 2 for frequency and extinction cross section), can be reconstructed using only four spectral parameters per QNM. In this way, the high‐dimensional spectral dataset is projected onto a low‐dimensional parameter space. Such reduction is particularly valuable in direct and inverse design of nanophotonic devices, where optimization and data‐driven approaches often suffer from the curse of dimensionality.

For interested readers who would like to verify the performance of our approach, we provide Python scripts and COMSOL files for several representative cases, including the computation of $q_{m}$ and $\sigma_{m}$ for a dispersive particle and particle on a substrate. These scripts are designed to be accessible to beginners in QNM theory. A more useful and advanced implementation will be available in the freeware MAN version 9 or later (available on Zenodo [27]). For details see Supporting Information S1: Section III.

## 4 Coupling‐Induced Changes of Fano Resonances: Case of Exceptional Points

According to Equations ((5a), (5b), (5c)) and (6), pronounced variations in $q_{m}$ can arise from substantial modifications of the QNM field ${\tilde{E}}_{m}$. Such changes commonly occur during QNM hybridization processes [34], for instance in the vicinity of quasi‐BICs [35]. Here, we instead focus on the more exotic and technically challenging regime of EPs [34, 43]. These non‐Hermitian singularities result from the coalescence of two photonic modes, and, to the best of our knowledge, the associated behavior of the Fano parameters has not previously been investigated within a fully analytical or QNM‐based framework.

We consider a ring resonator with a groove defect (inset in Figure 3a). In practice, operating precisely at an EP is unattainable, even in simulations. In the present case, the EP is obtained for a geometrical parameter, æ $=h_{d}/h\times 10^{3}\approx 14.39$, defined as the ratio between the groove depth and the ring height. For this value, the real and imaginary parts of eigenfrequency become equal within numerical accuracy (Figure 3a,b). Calculations of $q_{m}$ and $\sigma_{m}$ are performed with the methodology depicted in Figure 1. Their theoretical values were obtained with RFA for each value of æ.

**FIGURE 3:**
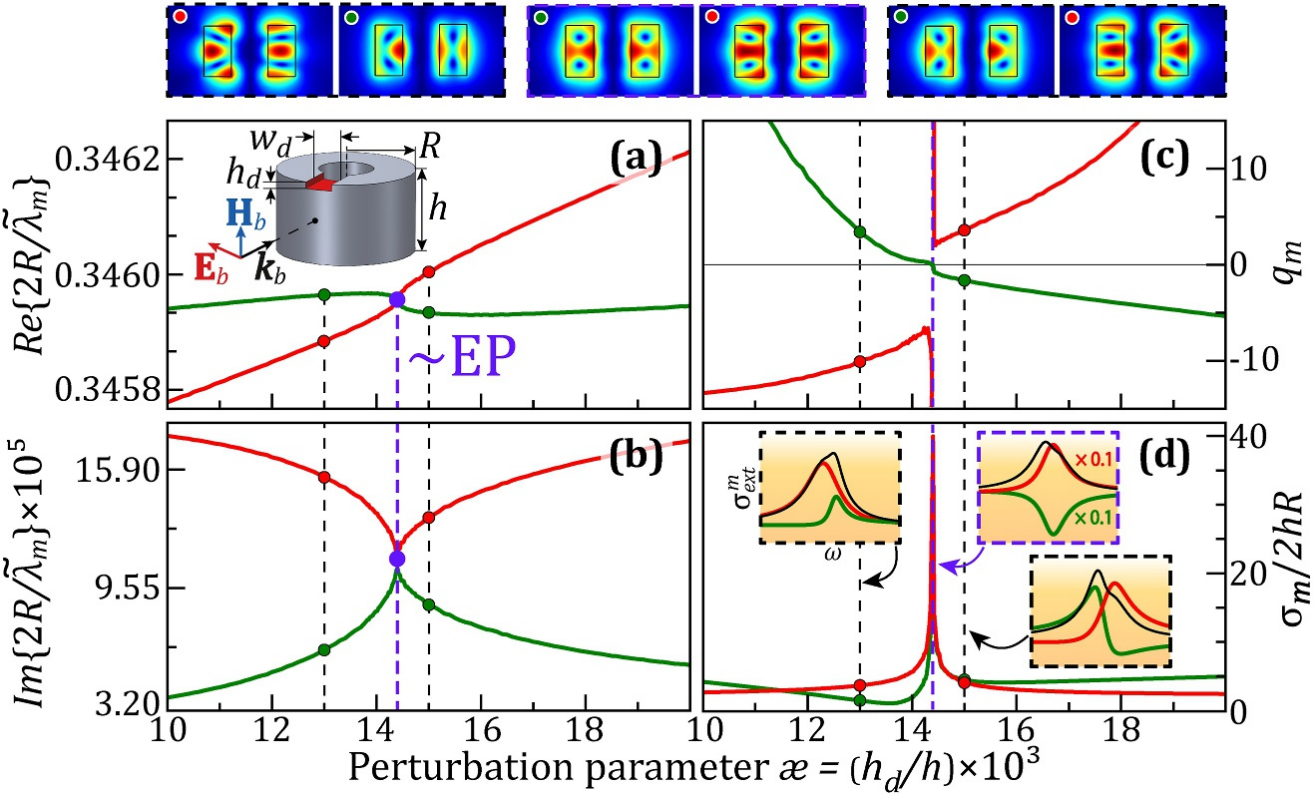
Complete characterization of spectral parameters near the EP. Top inset: distributions of $\left|{\tilde{E}}_{m}\right|$ for the QNMs for æ = 13, 14.39 (very close to EP), and 15, from left to right, respectively. View in the vertical mirror‐symmetry plane. (a–b) Real and imaginary parts of the resonance frequencies of the two coalescing QNMs as functions of the geometrical parameter æ ${=h}_{d}/h$ × 10^3^. (c–d) Evolution of the corresponding Fano parameters $q_{m}$ and $\sigma_{m}$ as æ varies. Inset in (a): sketch of a ring resonator with a small groove air defect highlighted in red. Parameters used in the computation are: ε= 43 + 0.003*i* for the ring, $\varepsilon_{b}=1$, r/R= 0.4, R/h= 0.894, R= 100 nm, $w_{d}=r$, where r is the inner ring radius, and $h_{d}$ is the variable. Insets in (d): Normalized extinction spectra (Equation 5a) for the same three values of æ. Red and green curves correspond to the two QNMs; black curves depict their sum. Note that, at æ = 14.39, the red and green resonances are very intense; they are scaled by 0.1 for better visualization. More details are found in Supporting Information S1: Section III.1.

Figure 3c,d illustrate the variation of the Fano parameters, $q_{m}$ and $\sigma_{m}$, for the two modes (hereafter labeled 1 and 2) as the control parameter æ is tuned across the EP. Both parameters exhibit singular behavior in the vicinity of the EP, driven by dramatic modifications of the QNMs field distributions (top panel in Figure 3). Specifically, the modal intensities of the two QNMs, $\sigma_{1}$ and $\sigma_{2}$, diverge, whereas their Fano factors tend to ±0 for the green mode and ∓∞ for the red one.

The divergence of $\sigma_{1}$ and $\sigma_{2}$ originates from the QNM normalization factor, which vanishes near EPs, leading to a blow‐up of the normalized fields as the modes coalesce. Importantly, QNM normalization carries deep physical meaning: it enables quantitative comparison between different modes and intrinsically incorporates the phase of the QNM fields, a quantity that plays a central role in fundamental light–matter interaction processes [44].

In particular, the phase of QNM norm is a key quantity for understanding the behavior of the Fano parameters in Figure 3c and, more generally, the behavior of the total spectral response in extinction cross‐section near an EP. In this regime, normalization constants of two coalescing modes are nearly out of phase, which leads to the approximate π/2 phase relation between *normalized* modal fields, namely ${\tilde{E}}_{1}\;\approx \;i{\tilde{E}}_{2}$ (Supporting Information S1: Section III.1).

Within the present QNM formalism, this phase relation implies $\xi_{1}\;\approx \;{\left(i\right)}^{2}\xi_{2}$, and hence $\sigma_{1}\;\approx \;\sigma_{2}$ and $q_{1}q_{2}\;\approx \;-1$. According to Equation (5a), exact fulfillment of these equalities, together with ${\tilde{\omega }}_{1}={\tilde{\omega }}_{2}$, would result in cancellation of the two resonant contributions and thus complete suppression of the total response. However, in the vicinity of an EP these equalities hold only approximately, leading to substantial but incomplete suppression. Specifically, Figure 3c,d) shows the emergence of such “opposite” Fano parameters in the limiting case $\left(q_{1},q_{2}\right)\;\approx \left(\pm \;0,\mp \;\infty \right)$.

Beyond elucidating the physics of Fano resonances in the vicinity of EPs, we emphasize two critical issues effectively addressed by the RFA approach. First, the majority of studies on Fano resonances in extinction or scattering spectra rely essentially on phenomenological fitting based on Equation (1) to extract line‐shape asymmetry and resonance intensity. However, this fitting procedure fails to accurately capture the spectral behavior associated with mode coalescence at an EP, where the responses of the two interacting modes fully merge with divergent and opposite contributions [34, 45] (insets in Figure 3d). Second, although EPs are widely regarded as promising building blocks for next‐generation optical devices, their signatures in total spectra, as shown above, are typically weak. Consequently, EPs are challenging to identify or exploit using spectral measurements alone—a limitation that is well‐recognized today [46, 47]. The RFA framework, by contrast, provides direct access to all spectral “markers” of photonic modes in the vicinity of EPs and thus represents a powerful and potentially indispensable tool for rendering EPs spectroscopically distinguishable.

## 5 Tuning Fano Resonances via the Incident Field

Following Equations ((5a), (5b), (5c)) and (6), an alternative way to vary $q_{m}$ is to vary the incident field $E_{b}$. This mechanism validated through comparison between RFA and experimental results. We consider a split‐ring resonator with a rectangular cross‐section, fabricated from a high‐index low‐loss ceramic compound, (Ca_0,67_La_0,33_) (Al_0,33_Ti_0,67_)O_3_, which has a relative permittivity, ε = 43 + 0.0034i, nearly constant across the 1–8 GHz frequency range used in the experiment. A photograph of the split‐ring is shown in Figure 4a.

**FIGURE 4:**
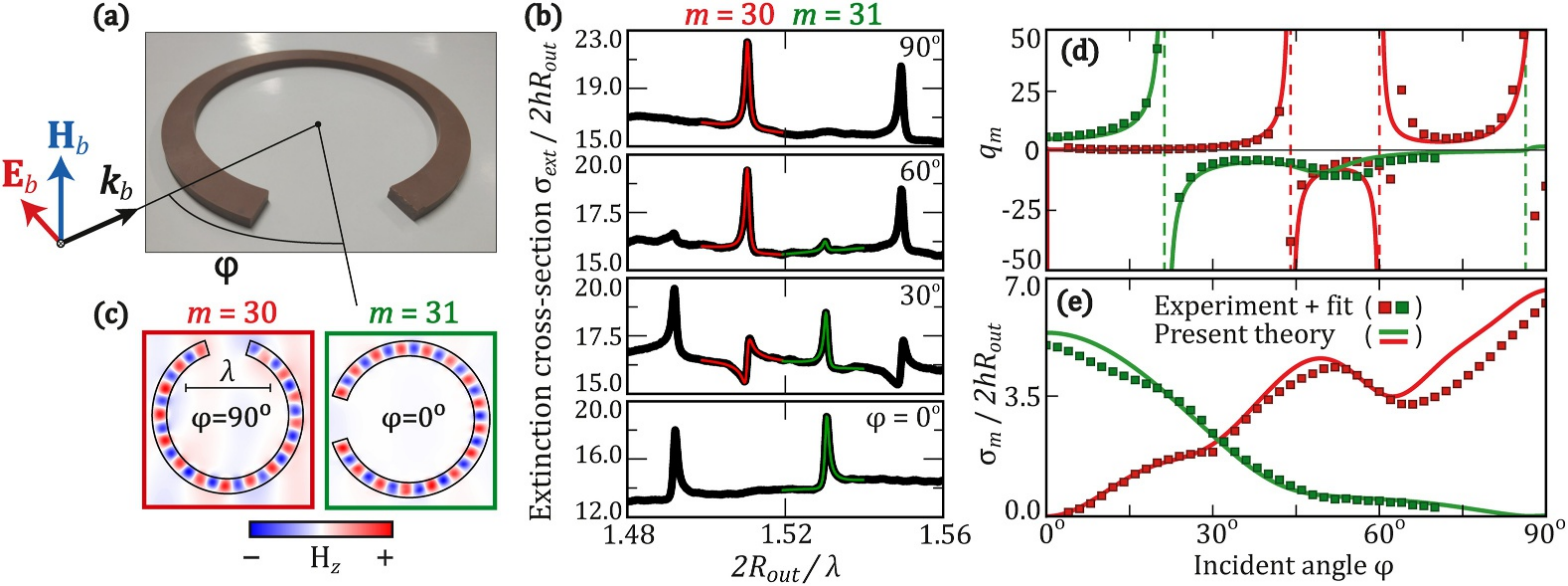
Comparison between the QNM Fano theory and the classical phenomenological approach based on a fit of experimental spectra. (a) Photograph of the split‐ring used in the experiment. (b) Measured extinction spectra for several incident angles φ with superimposed green and red lines representing fits using Equation (1). (c) Near‐field measurements of the magnetic fields of the anti‐symmetric m= 30 and symmetric m= 31 modes. The scale bar indicates the incident wavelength in air at $2R_{\text{out}}/\lambda =$ 1.51. (d) and (e) Comparison between theory (Equations ((5a), (5b), (5c)) and (6), RFA) and experiment (Fano fit with Equation 1) for the Fano parameter $q_{m}$ and resonance intensity $\sigma_{m}$. Vertical dashed lines in (d) indicate the theoretical positions of $q_{m}=0,\pm \infty$. The resonator parameters are: a height‐to‐width ratio $h/\left(R_{\text{out}}-R_{\text{in}}\right)=$ 0.36, inner and outer radius ratio $R_{\text{in}}/R_{\text{out}}=$ 0.81 with $R_{\text{out}}=$ 57.5 mm, and a half‐gap angular size β= 18°. Note the color labeling consistently used in all panels: m= 30 (red) and 31 (green).

To measure the extinction spectrum, we place the split‐ring between two horn antennas positioned 2 m apart in an anechoic chamber (see details in Supporting Information S1: Section IV). The spectrum was inferred by comparing the $S_{21}$ measurements with and without the sample. The broken rotational symmetry of the split‐ring offers an ideal way to study the Fano parameter for different orientations φ between the sample and the incident light (Figure 4a). Figure 4b displays four extinction spectra measured for φ= 0°, 30°, 60°, and 90°, which, in agreement with Equations ((5a), (5b), (5c)) and (6), demonstrates that Fano parameters are strongly dependent on the background field.

The resonator supports longitudinal Fabry‐Pérot‐like resonances, indexed by the number m of half‐wavelengths fitting within the optical length of the resonator. Figure 4c shows the near‐field distributions recorded at resonance for m= 30 (red) and 31 (green), approximately 2 mm above the split‐ring, using a magnetic near‐field probe. The two modes exhibit different symmetries.

The measured spectra were fitted using the classical Fano formula (Equation 1), including an additional background intensity term $\sigma_{bg}$, approximated by a second‐order polynomial of the frequency. The red and green curves in Figure 4b illustrate the high accuracy of the fit. The fitted parameters, $q_{m}$ and $\sigma_{m}$, are presented in the square markers in Figure 4d,e, respectively. Corresponding theoretical values of $q_{m}$ and $\sigma_{m}$ for each φ were obtained with RFA (solid curves in Figure 4d,e). Since $E_{b}$ is defined analytically, we obtain the latter dependencies with high angular resolution and almost instantly—for the angular step Δφ=0.045° the computational time for integrals is less than 5 s. The single QNM computation required nearly 2 min.

A strong quantitative agreement between the fitted values of $q_{m}$ and $\sigma_{m}$ (square markers) and the theoretical predictions (solid curves) is achieved for both modes, over a wide range of $q_{m}$ and $\sigma_{m}$ values and for all incident angles φ. The minor deviation observed around φ= 60° is caused by experimental noise, as shown in Supporting Information S1: Section III.2 with numerical frequency‐domain simulations of the split‐ring extinction.

Overall, the q‐curves for both modes in Figure 4d exhibit distinctive transitions from $q_{m}=+\infty$ to $q_{m}=-\infty$ at specific values of φ, where $\arg\left(\xi_{m}\right)/2=$ 0 (Equation 5b). Conversely, $|\arg\left(\xi_{m}\right)|/2$ remains close to π/2 at small angles φ < 15° for m= 30 or at large angles φ > 75° for m= 31, leading to small absolute values of Fano parameters. This behavior results from vanishing resonance intensity (Figure 4e) due to the different symmetries of the two modes.

Such complicated variations in the Fano parameter occur exclusively for high‐order modes, where the resonator size exceeds the incident wavelength, causing different parts of the mode to interact with varying phases of the incident wave (Figure 4c). In contrast, for low‐order modes, this mechanism is negligible because $E_{b}$ is nearly uniform throughout the resonator volume. In such cases, only variations in the intensity $\sigma_{m}$ may arise due to symmetry protection, whereas $q_{m}$ does not exhibit significant changes [48, 49].

## 6 Applicability of Resonant Frequency Approximation for Dispersive Nanostructures

Earlier analytical expressions for Fano parameters [35, 42] were constrained to idealized systems, limiting their relevance for practical photonic designs. In this section, we demonstrate that the present RFA framework is equally applicable for realistic optical nanostructures, accommodating material dispersion, substrate effects, and arbitrary excitation conditions.

We consider a system composed of two Ag nanocubes with side length a= 65 nm and separated by a gap g= 10 nm. The nanoparticles are placed on an Ag‐substrate covered with an 8 nm‐thick spacer. They are illuminated by a TM‐polarized plane wave at oblique incidence, mimicking conditions in standard dark‐field experiments (insert in Figure 5). The dispersive properties of Ag are described with the Drude model $\varepsilon_{Ag}=\varepsilon_{\infty }-\omega_{p}^{2}/\omega \left(\omega +i\gamma \right)$ (for details see Supporting Information S1: Section II.1), whereas the spacer is treated as a non‐dispersive material with refractive index $n_{sp}=$ 1.5. For the calculations, we employ the QNM auxiliary‐field formulation [26], incorporated in the freeware “MAN” [27], to account for the dispersive properties along with Equations ((5a), (5b), (5c)) and (6) with RFA. The RFA results are compared with the “exact” standard frequency domain COMSOL calculation.

**FIGURE 5:**
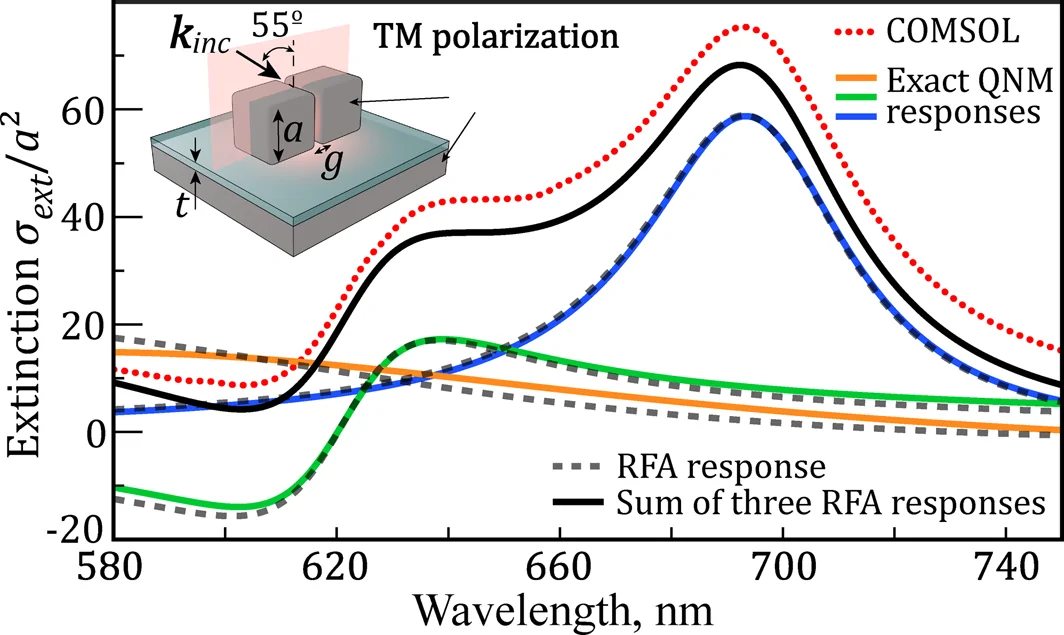
Demonstration of the RFA performance in the case of the plasmonic nanostructure: two dispersive silver nanocubes placed on a layered substrate. Extinction cross section calculated with the standard COMSOL solver (reference “exact” data) represented with red dotted curve. Dark dashed curves correspond to RFA (only analytical $\omega^{2}-$dependence of $\sigma_{m}$ is preserved), colored curves to “exact” QNM responses (all ω−dependencies are preserved). Geometrical parameters shown in the insert: a= 65 nm; g= 10 nm; t= 8 nm. Permittivity of Ag is described with the Drude model, the non‐dispersive spacer has refractive index $n_{sp}=$ 1.5. Note that $E_{b}=E_{\text{inc}}+E_{\text{ref}}$, where $E_{\text{ref}}$ is the reflected wave obtained with Fresnel equations for two interfaces.

As shown in Figure 5, the RFA reconstruction agrees closely with both the individual “exact” QNM contributions and the total extinction obtained from the standard COMSOL computation. Only three QNMs contribute appreciably, which is typical of plasmonic nanostructures. The two dominant modes (blue and green curves with Q= 15.3 and 17.0) are well‐reproduced with constant $q_{m}$ and $\left|\xi_{m}\right|$. A slight decrease in accuracy is observed for the third mode (orange curve with Q=2.3), mainly due to Ag dispersion, as discussed in Supporting Information S1: Section II.1; such low‐Q modes are typically of limited interest in practice. Overall, the results in Figure 5 provide clear evidence that the RFA framework is well suited for realistic optical nanostructures.

A common goal in nanoparticle design is to design extinction spectra with specific features. However, numerically optimizing continuous objectives can be inconvenient, unreliable, and computationally expensive—especially for nanoparticles embedded in heterogeneous environments. In these applied problems, the RFA method proves to be highly effective, as it operates with the four key parameters—${\omega_{m},\gamma_{m},q}_{m},\left|\xi_{m}\right|$ or $\sigma_{m}$—that can be directly integrated into various optimization algorithms, including gradient descent and others. A simple example of such optimization is presented in Supporting Information S1: Section III.6 (with used [50, 51]), where we demonstrate that RFA can be used to optimize the detection limits of optical resonance sensors by tuning the geometry and adjusting the incident beam direction in a very effective way.

As a final remark, Equations ((5a), (5b), (5c)) and (6) for the Fano parameters are derived without approximations. This implies that even in the presence of complex interactions caused by background medium or strong material dispersion, electromagnetic resonances will retain a Fano‐type profiles in extinction spectra. We mentioned earlier that different mechanisms of light‐matter interaction are encoded in the permittivity contrast Δε(ω,r), which determines the eigenstates ${\tilde{E}}_{m}$ along with the geometry. The permittivity contrast can include material poles, which represent challenging scenario for modern numerical methods. Nevertheless, in Supporting Information S1: Section III.7 (with used [52, 53, 54, 55]), we present an example involving a perovskite nanoparticle on a substrate, over a frequency range that includes the intrinsic material resonance of the perovskite. Despite additional complications, the RFA still performs well, illustrating the generality and robustness of the proposed method.

## 7 Conclusion

This work positions QNM theory of scattering as a central element in the broader family of “Fano effects,” which influence many areas of physics, including various contemporary photonic problems with completely different decay physics, such as material absorption or leakage through various radiative channels. It presents QNMs as a unifying framework that integrates discrete and continuum states, by encapsulating critical information about the phases of both narrow and broad resonances all in one. This perspective offers a refreshing alternative to conventional coupled oscillator models of Fano resonances.

Beyond their broad theoretical scope, the expressions for the Fano asymmetry parameter q and intensity σ in Equations ((5a), (5b), (5c)) and (6) are both simple and physically sound. Calculating these parameters requires only the normalized QNM fields, which are readily accessible through modern QNM solvers. This simplicity enables rapid optimization of resonator geometries and illumination conditions. Moreover, the phase properties of singular features in photonics, such as EPs, can be studied precisely with the present theory up to numerical accuracy.

More generally, the explicit and general formulas for Fano parameters introduced here holds great promise for substantially accelerating spectral calculations and fostering new advances in photonic device engineering and analysis. When combined with computational‐bounds approaches [56], QNM formalisms for inverse design [57, 58], or deep learning techniques [59, 60, 61], the present framework is poised to catalyze the next generation of spectral response design and optimization in photonics.

## Author Contributions

**Mikhail Bochkarev:** investigation, conceptualization, writing – review and editing, methodology. **Nikolay Solodovchenko** **:** investigation, conceptualization, methodology. **Kirill Samusev:** methodology. **Mikhail Limonov:** supervision, writing – review and editing. **Tong Wu:** methodology, writing – review and editing. **Philippe Lalanne:** supervision, methodology, conceptualization, writing – original draft.

## Funding

P. Lalanne acknowledges financial support from the UPiCO project (ANR‐25‐CE09‐4019) and the European Union's Horizon research and innovation program under the Marie Skłodowska‐Curie (QUARTE project, 101148330).

## Conflicts of Interest

The authors declare no conflicts of interest.

## Supporting information


Supporting Information S1

## Data Availability

Data underlying the results presented in this paper are available in [27] and supplementary material. Additional data related to this paper are available from the co‐first authors upon request.
