# Supplementary material for: Electromagnetic Quasinormal Modes: A Foundational Framework for Describing Fano Resonances of Extinction Spectra
Source: Nanophotonics. 2026 Apr 25;15(9):e70071. doi: 10.1002/nap2.70071 (PMC13642088; doi:10.1002/nap2.70071)
Supplement: Supplementary file 1 [file NAP2-15-e70071-s001.pdf]

Supplemental Document

Electromagnetic quasinormal modes: a foundational framework for describing Fano resonances of extinction spectra

Mikhail Bochkarev,[1,2†] Nikolay Solodovchenko,[1,2†], Kirill Samusev,[1,2], Mikhail Limonov,[1,2] Tong Wu,[3] and Philippe Lalanne[3]

[1]*School of Physics and Engineering, ITMO University, Saint-Petersburg, 191002, Russia*
[2]*Ioffe Institute, Saint-Petersburg, 194021, Russia*
[3]*Laboratoire Photonique Numérique et Nanosciences (LP2N), Université de Bordeaux, Institut d'Optique Graduate School, CNRS, Talence, France*
[†]*Co-first authors with equal contribution*

OUTLINE

I. Quasinormal mode theory for Fano resonances 2
I.1 Derivation of the equations for q_m and σ_m 2
I.2 Geometrical representation of the Fano parameter 5
II. Applicability of the Resonant frequency approximation (RFA) 6
II.1 Accuracy of RFA reconstruction 6
II.2 Frequency sampling and computational cost analysis 8
III. Performance of the theory: Demonstration for a few different cases 11
III.1 Exceptional points in ring resonator with defect 11
III.2 Resonances in single dielectric split-ring resonator 14
III.3 Scattering from a dielectric cylinder offering a bound-state in the continuum 14
III.4 Dielectric resonator in a hollow metallic waveguide 16
III.5 Scattering from a dielectric split-ring resonator on a substrate 16
III.6 Plasmonic nanoresonator on a substrate. Optical sensor optimization 17
III.7 Dispersive perovskite nanoparticle on layered substrate 19
IV. Experimental setup 22
References to supplemental document 23

I. Quasinormal mode theory for Fano resonances

I.1 Derivation of the equations for q_m and σ_m

In this section, we derive Eqs. (5) and (6) from the main text, following the sign convention $e^{-i\omega t}$ used in the main text. Since a key objective of this work is to bridge quasinormal modes (QNMs) and Fano response with the highest level of generality, a detailed step-by-step derivation will be provided to highlight that the correspondence is obtained without any approximations. Ultimately, we will see that only a single simplification is involved. The general equation for the extinction cross-section is [1],[2]

$$\sigma_{ext} = \frac{1}{P_0}\frac{\omega}{2}\int_{V_r} Im\{\varepsilon_0(\varepsilon-\varepsilon_b)(\mathbf{E}_s+\mathbf{E}_b)\cdot\mathbf{E}_b^*\}\,d^3\boldsymbol{r}$$
$$+\frac{1}{P_0}\frac{\omega}{2}\int_{V_r} \varepsilon_0 Im\{\varepsilon_b\}[|\mathbf{E}_s+\mathbf{E}_b|^2-|\mathbf{E}_s|^2]\,d^3\boldsymbol{r}, \tag{S1}$$

where ε is resonator's permittivity, which can be a dispersive tensor, and ε_b is permittivity of surrounding media; $\mathbf{E}_s$ is the electric field scattered by the resonator upon illumination by the background electric field $\mathbf{E}_b$; P_0 is the time-averaged Poynting vector.

For non-absorbing host media ($Im\{\varepsilon_b\}=0$) and the Drude-Lorentz permittivity of the resonator $\varepsilon = \varepsilon_\infty - \varepsilon_\infty\omega_p^2/(\omega^2-\omega_0^2+i\omega\gamma) \equiv \varepsilon_\infty + \varepsilon_{DL}(\omega)$, Eq. (S1) transforms into

$$\sigma_{ext} = \frac{1}{P_0}\frac{\omega}{2}\int_{V_r} Im\{\varepsilon_0(\varepsilon_\infty+\varepsilon_{DL}(\omega)-\varepsilon_b)(\mathbf{E}_s+\mathbf{E}_b)\cdot\mathbf{E}_b^*\}\,d^3\boldsymbol{r} =$$
$$= \frac{1}{2P_0}\int_{V_r} Im\{\omega\varepsilon_0(\varepsilon_\infty-\varepsilon_b)\mathbf{E}_s\cdot\mathbf{E}_b^* + i\mathbf{J}_{tot}\cdot\mathbf{E}_b^*\}\,d^3\boldsymbol{r}$$
$$+\frac{\omega}{2P_0}\int_{V_r} Im\{\varepsilon_0(\varepsilon_\infty-\varepsilon_b)\mathbf{E}_b\cdot\mathbf{E}_b^*\}\,d^3\boldsymbol{r}, \tag{S2}$$

where $\mathbf{J}_{tot} \equiv -i\omega\boldsymbol{P}_{tot} = -i\omega\varepsilon_0\varepsilon_{DL}(\omega)(\mathbf{E}_s+\mathbf{E}_b)$ is the total polarization current. Note that for generality we consider $\varepsilon_\infty \in \mathbb{C}$, which may be necessary in the case of a non-dispersive material with losses.

The single simplification ($Im\{\varepsilon_b\}=0$) is applied for the convenience of subsequent mathematical derivation, but it is not fundamentally necessary; also note that the Drude-Lorentz permittivity can be considered with several poles. The scattered field $\mathbf{E}_s$ can be represented with a QNM expansion

$$\mathbf{E}_s = \sum_m \alpha_m(\omega)\tilde{\mathbf{E}}_m(r) + \sum_k \alpha_k^{(Num)}(\omega)\tilde{\mathbf{E}}_k^{(Num)}(r), \tag{S3}$$

with expansion coefficient (known as excitation coefficient), according to [3]

$$\alpha_m(\omega) = \frac{\tilde{\omega}_m}{\tilde{\omega}_m-\omega}\int_{V_r} \varepsilon_0(\varepsilon(\tilde{\omega}_m)-\varepsilon_b)\tilde{\mathbf{E}}_m\cdot\mathbf{E}_b d^3\boldsymbol{r} + \int_{V_r}\varepsilon_0(\varepsilon_b-\varepsilon_\infty)\tilde{\mathbf{E}}_m\cdot\mathbf{E}_b d^3\boldsymbol{r}$$
$$= -\frac{\omega}{\omega-\tilde{\omega}_m}\int_{V_r}\varepsilon_0\left(\varepsilon_\infty+\left(\frac{\tilde{\omega}_m}{\omega}\right)\varepsilon_{DL}(\tilde{\omega}_m)-\varepsilon_b\right)\tilde{\mathbf{E}}_m\cdot\mathbf{E}_b d^3\boldsymbol{r}$$
$$\equiv -\frac{\omega}{\omega-\tilde{\omega}_m}\int_{V_r}\Delta\varepsilon\tilde{\mathbf{E}}_m\cdot\mathbf{E}_b d^3\boldsymbol{r} \tag{S4}$$

where $\tilde{\omega}_m = \omega_m - i\gamma_m$ is the complex resonant frequency of the mth mode; the dielectric contrast $\Delta\varepsilon \equiv \varepsilon_0(\varepsilon_\infty + (\tilde{\omega}_m/\omega)\varepsilon_{DL}(\tilde{\omega}_m) - \varepsilon_b)$, with dispersive part ε_{DL} that must be taken at complex eigenfrequency $\tilde{\omega}_m$.

In the expansion Eq. (S3), we have introduced numerical modes (or PML-modes) which are necessary to have a complete basis in a case where resonators are not in free space (i.e. for the most general case involving non-uniform layered substrates), but in the derivation below they are not considered (note that $\alpha_k^{(Num)}$ is the same as in Eq. (S4), the index "Num" highlights only the numerical nature of the PML-modes).

According to [3], the polarization current $\mathbf{J}_{tot}$ can be represented with the next expression

$$\mathbf{J}_{tot} = \sum_m \alpha_m \tilde{\mathbf{J}}_m = -i \sum_m \tilde{\omega}_m \alpha_m \tilde{\mathbf{P}}_m = -i \sum_m \tilde{\omega}_m \varepsilon_0 \varepsilon_{DL}(\tilde{\omega}_m) \alpha_m \tilde{\mathbf{E}}_m. \quad \text{(S5)}$$

Thus, the extinction cross section (Eq. (S2)) of all the modes can be written

$$\begin{aligned}
\sigma_{ext} &= \frac{1}{2P_0}\int_{V_r} Im\{\omega\varepsilon_0(\varepsilon_\infty - \varepsilon_b)\mathbf{E}_b^* \cdot \mathbf{E}_s + i\mathbf{E}_b^* \cdot \mathbf{J}_{tot}\} + \frac{\omega}{2P_0}\int_{V_r} Im\{\varepsilon_0(\varepsilon_\infty - \varepsilon_b)\mathbf{E}_b \cdot \mathbf{E}_b^*\}\, d^3\boldsymbol{r} \\
&= \frac{1}{2P_0}\int_{V_r} Im\{\omega\varepsilon_0(\varepsilon_\infty - \varepsilon_b)\mathbf{E}_b^* \cdot \sum_m \alpha_m \tilde{\mathbf{E}}_m + i\mathbf{E}_b^* \cdot (-i)\sum_m \tilde{\omega}_m \varepsilon_0 \varepsilon_{DL}(\tilde{\omega}_m)\alpha_m \tilde{\mathbf{E}}_m\} d^3\boldsymbol{r} \\
&+ \frac{\omega}{2P_0}\int_{V_r} Im\{\varepsilon_0(\varepsilon_\infty - \varepsilon_b)\mathbf{E}_b \cdot \mathbf{E}_b^*\}\, d^3\boldsymbol{r} \\
&= \frac{\omega}{2P_0}\int_{V_r} Im\{\sum_m \varepsilon_0(\varepsilon_\infty + (\tilde{\omega}_m/\omega)\varepsilon_{DL}(\tilde{\omega}_m) - \varepsilon_b)\alpha_m \tilde{\mathbf{E}}_m \cdot \mathbf{E}_b^*\} d^3\boldsymbol{r} \\
&+ \frac{\omega}{2P_0}\int_{V_r} Im\{\varepsilon_0(\varepsilon_\infty - \varepsilon_b)\mathbf{E}_b \cdot \mathbf{E}_b^*\}\, d^3\boldsymbol{r} \\
&= \frac{\omega}{2P_0} Im\left\{\sum_m \left(-\frac{\omega}{\omega - \tilde{\omega}_m}\right)\left(\int_{V_r} \Delta\varepsilon \tilde{\mathbf{E}}_m \cdot \mathbf{E}_b d^3\boldsymbol{r}'\right)\left(\int_{V_r} \Delta\varepsilon \tilde{\mathbf{E}}_m \cdot \mathbf{E}_b^* d^3\boldsymbol{r}\right)\right\} \\
&+ \frac{\omega}{2P_0}\int_{V_r} Im\{\varepsilon_0(\varepsilon_\infty - \varepsilon_b)\mathbf{E}_b \cdot \mathbf{E}_b^*\}\, d^3\boldsymbol{r}. \quad \text{(S6)}
\end{aligned}$$

The last term in Eq. (S6) does not depend on the mode. It merely adds a uniform background extinction, when $\varepsilon_\infty \in \mathbb{C}$

$$\sigma_{ext}^{bg} \equiv \frac{\omega}{2P_0} Im\left\{\left(\int_{V_r} \varepsilon_0(\varepsilon_\infty - \varepsilon_b)\mathbf{E}_b^* \cdot \mathbf{E}_b\, d^3\boldsymbol{r}\right)\right\}. \quad \text{(S7)}$$

More interestingly, let us consider the other term in Eq. (S6), which represents the extinction produced by the mth QNM:

$$\sigma_{ext}^{m} = \frac{\omega}{2P_0} Im\left\{\left(-\frac{\omega}{\omega - \tilde{\omega}_m}\right)\left(\int_{V_r} \Delta\varepsilon \tilde{\mathbf{E}}_m \cdot \mathbf{E}_b d^3\boldsymbol{r}'\right)\left(\int_{V_r} \Delta\varepsilon \tilde{\mathbf{E}}_m \cdot \mathbf{E}_b^* d^3\boldsymbol{r}\right)\right\}. \quad \text{(S8)}$$

We now introduce the key parameter of our work

$$\xi_m \equiv \left(\int_{V_r} \Delta\varepsilon \tilde{\mathbf{E}}_m \cdot \mathbf{E}_b d^3\boldsymbol{r}'\right)\left(\int_{V_r} \Delta\varepsilon \tilde{\mathbf{E}}_m \cdot \mathbf{E}_b^* d^3\boldsymbol{r}\right), \quad \text{(S9)}$$

and further introduce the following notation for convenience

$$\Omega_m \equiv \frac{\omega - \omega_m}{\gamma_m} \in \mathbb{R}\,. \quad \text{(S10)}$$

Using the identity

$$\left(-\frac{\omega}{\omega-\tilde{\omega}_m}\right) = -\left(\frac{\omega}{\omega-(\omega_m-i\gamma_m)}\right) = -\frac{\omega}{\gamma_m}\left(\frac{1}{\frac{\omega-\omega_m}{\gamma_m}+i}\right) = -\frac{\omega}{\gamma_m}\left(\frac{\Omega_m-i}{\Omega_m^2+1}\right), \tag{S11}$$

we obtain

$$\begin{aligned}\sigma_{ext}^m &= \frac{\omega}{2P_0} Im\left\{-\frac{\omega}{\gamma_m}\left(\frac{\Omega_m-i}{\Omega_m^2+1}\right)\xi_m\right\} = -\frac{1}{P_0}\frac{\omega^2}{2\gamma_m}\left(\frac{1}{\Omega_m^2+1}\right) Im\{(\Omega_m-i)\xi_m\} \\ &= -\frac{1}{P_0}\frac{\omega^2}{2\gamma_m}\left(\frac{1}{\Omega_m^2+1}\right)\left[\Omega_m Im\{\xi_m\} - Re\{\xi_m\}\right] \\ &= \frac{1}{P_0}\frac{\omega^2}{2\gamma_m}\left(\frac{1}{\Omega_m^2+1}\right) Re\{\xi_m\}\left[\frac{\Omega_m}{\left(-\frac{Re\{\xi_m\}}{Im\{\xi_m\}}\right)}+1\right].\end{aligned} \tag{S12}$$

Now we introduce, by definition, two quantities

$$\psi \equiv -\frac{Re\{\xi_m\}}{Im\{\xi_m\}}, \tag{S13}$$

$$q_m \equiv \psi + \sqrt{\psi^2+1} = -\frac{Re\{\xi_m\}}{Im\{\xi_m\}} \pm \frac{\sqrt{Re\{\xi_m\}^2+Im\{\xi_m\}^2}}{|Im\{\xi_m\}|} = -\frac{Re\{\xi_m\}+|\xi_m|}{Im\{\xi_m\}}. \tag{S14}$$

In Eq. (S14), we select the negative sign for the term $\pm|Im\{\xi_m\}|$. This choice is based on the comparison between the final theoretical and numerical results. It's worth noting that the form $q_m = \psi + \sqrt{\psi^2+1}$ was previously obtained in quantum mechanics [4]. We also point out that this choice of q_m corresponds to the classical form of the Fano parameter (see the next section I.2).

Introducing a simple algebraic relation, we have

$$\frac{1}{\psi} = \frac{1}{\psi}\left(\frac{2\psi+2\sqrt{\psi^2+1}}{2\psi+2\sqrt{\psi^2+1}}\right) = 2\left(\frac{\psi+\sqrt{\psi^2+1}}{\psi^2+2\psi\sqrt{\psi^2+1}+(\psi^2+1)-1}\right) = 2\left(\frac{\psi+\sqrt{\psi^2+1}}{\left(\psi+\sqrt{\psi^2+1}\right)^2-1}\right) = \frac{2q_m}{q_m^2-1} \tag{S15}$$

and applying this result to Eq. (S12), we obtain

$$\begin{aligned}\sigma_{ext}^m &= \frac{1}{P_0}\frac{\omega^2}{2\gamma_m}\left(\frac{1}{\Omega_m^2+1}\right) Re\{\xi_m\}\left[\frac{\Omega_m}{\psi}+1\right] = \frac{1}{P_0}\frac{\omega^2}{2\gamma_m}\left(\frac{1}{\Omega_m^2+1}\right) Re\{\xi_m\}\left[\frac{2q_m\Omega_m}{q_m^2-1}+1\right] \\ &= \frac{1}{P_0}\frac{\omega^2}{2\gamma_m}\left(\frac{1}{\Omega_m^2+1}\right) Re\{\xi_m\}\left[\frac{q_m^2-1+2q_m\Omega_m}{q_m^2-1}\right] \\ &= \frac{1}{P_0}\frac{\omega^2}{2\gamma_m}\left(\frac{1}{\Omega_m^2+1}\right) Re\{\xi_m\}\left(\frac{q_m^2+1}{q_m^2-1}\right)\left[\frac{q_m^2-1+2q_m\Omega_m}{q_m^2+1}\right]\end{aligned} \tag{S16}$$

We will consider the term $Re\{\xi_m\}\left(\frac{q_m^2+1}{q_m^2-1}\right)$ in detail. Using the definitions of q_m and ψ from Eqs. (S13) and (S14), it is straightforward to show that

$$q_m^2+1 = \left(-\frac{Re\{\xi_m\}+|\xi_m|}{Im\{\xi\}}\right)^2+1 = 2|\xi_m|\left(\frac{Re\{\xi_m\}+|\xi_m|}{Im^2\{\xi_m\}}\right), \tag{S17}$$

$$q_m^2-1 = \left(-\frac{Re\{\xi_m\}+|\xi_m|}{Im\{\xi_m\}}\right)^2-1 = 2Re\{\xi\}\left(\frac{Re\{\xi_m\}+|\xi_m|}{Im^2\{\xi_m\}}\right). \tag{S18}$$

Substituting Eqs. (S17) and (S18) into the term yields a very natural result

$$Re\{\xi_m\}\left(\frac{q_m^2+1}{q_m^2-1}\right) = |\xi_m|. \tag{S19}$$

Finally, we find that the QNM contribution to the extinction precisely follows a Fano line shape

$$\sigma_{ext}^{m}=\frac{1}{P_0}\frac{\omega^2}{2\gamma_m}|\xi_m|\left[\frac{\left(-\frac{Re\{\xi_m\}+|\xi_m|}{Im\{\xi_m\}}\right)^2-1+2\left(-\frac{Re\{\xi_m\}+|\xi_m|}{Im\{\xi_m\}}\right)\Omega_m}{(\Omega_m^2+1)\left(\left(-\frac{Re\{\xi_m\}+|\xi_m|}{Im\{\xi_m\}}\right)^2+1\right)}\right]=\sigma_m\left[\frac{q_m^2-1+2q_m\Omega_m}{(\Omega_m^2+1)(q_m^2+1)}\right], \tag{S20}$$

with

$$\Omega_m\equiv\frac{\omega-\omega_m}{\gamma_m}\in\mathbb{R}, \tag{S10}$$

$$q_m=-\frac{Re\{\xi_m\}+|\xi_m|}{Im\{\xi_m\}}, \tag{S21}$$

$$\sigma_m=\frac{1}{P_0}\frac{\omega^2}{2\gamma_m}|\xi_m|, \tag{S22}$$

$$\xi_m\equiv\left(\int_{V_r}\Delta\varepsilon\tilde{\mathbf{E}}_m\cdot\mathbf{E}_b d^3\mathbf{r}'\right)\left(\int_{V_r}\Delta\varepsilon\tilde{\mathbf{E}}_m\cdot\mathbf{E}_b^* d^3\boldsymbol{r}\right), \tag{S9}$$

where we repeat Eqs. (S9) and (S10) for the sake of clarity; let us also recall that in Eq. (S9) the dielectric contrast is a function of frequency and eigenfrequency: $\Delta\varepsilon\equiv\varepsilon_0(\varepsilon_\infty+(\tilde{\omega}_m/\omega)\varepsilon_{DL}(\tilde{\omega}_m)-\varepsilon_b)$. This result is established **without any approximations** for Drude-Lorentz materials; however, it can be adapted for N-pole permittivity model [5].

In Eq. (S20), the term in square brackets is dimensionless (this is the shape function), while the term outside the brackets, σ_m, has dimension of L^2. Further note that we used normalized QNMs and the normalization constant has dimensions of the square root of energy. As a result, in Eqs. (S3-S4), the excitation coefficient $\alpha_m(\omega)$ is dimensionless and the term σ_m has dimension of L^2. The term ξ_m has dimension of energy.

I.2 Geometrical representation of the Fano parameter

In this subsection, we relate Eq. (S21) to the traditional cotangent form for the Fano parameter using basic geometric calculus. Figure S1 provides a geometrical illustration in the complex plane $Real(\xi_m)-Imag(\xi_m)$.

We note that angles $\theta^{(1)}$ and $\theta^{(2)}$ are equal because the orange secant line intersects two parallel horizontal lines. Additionally, the geometry is designed such that the purple dashed triangle is isosceles, which implies $\theta^{(2)}=\theta^{(3)}$ and $\Delta=2\theta=\arg(\xi_m)$. Moreover, the angle θ is connected to Eq. (S21) through the relationship $\cot(\theta)=(Re\{\xi_m\}+|\xi_m|)/Im\{\xi_m\}=-q_m$.

With this result, we ultimately derive an expression that aligns with the conventional form of the Fano parameter. This expression involves the argument of ξ_m

$$q_m=-\frac{Re\{\xi_m\}+|\xi_m|}{Im\{\xi_m\}}=-\cot(\theta)=-\cot\left(\frac{\Delta}{2}\right)=-\cot\left(\frac{\arg(\xi_m)}{2}\right). \tag{S23}$$

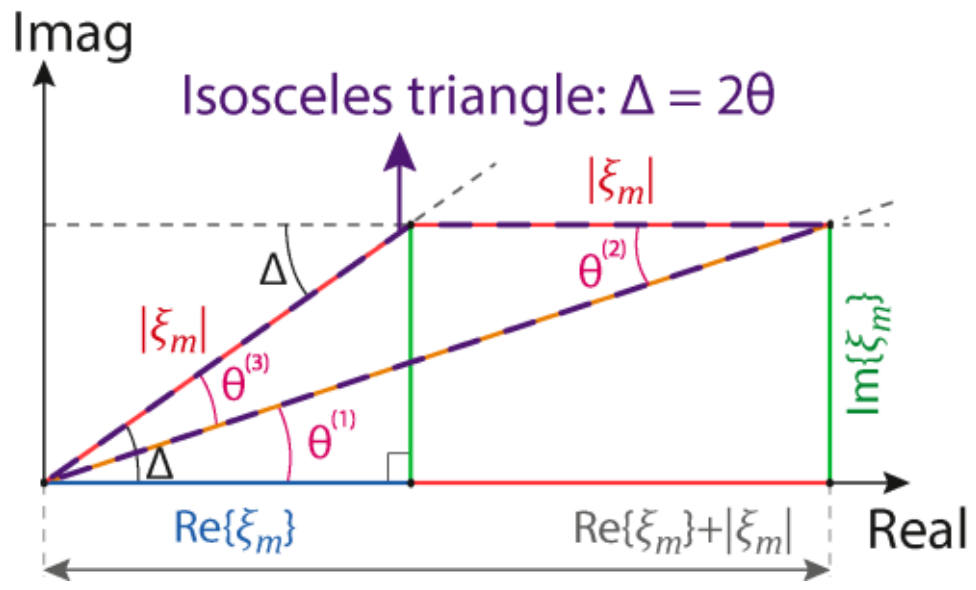

Fig. S1 – Geometrical representation of the Fano parameter.

II. Applicability of the Resonant frequency approximation (RFA)

The main text emphasizes that both q_m and σ_m (Eqs. S21 and S22) are frequency-dependent functions. However, it is convenient to disregard this dependence and treat these parameters as constant values. This simplification can be made under the "resonant frequency approximation" (RFA), the validity of which will be thoroughly tested in the following subsections.

II.1 Accuracy of RFA reconstruction

Test of the RFA. We consider a spherical particle with the diameter $D = 50$ mm and neglect the dispersion, $\varepsilon = 16$, around the resonant frequency. We compare results for the exact scattering (extinction) responses of QNMs obtained with Eq. (S20), with RFA responses, for which q_m and σ_m are obtained with Eqs. (S21) and (S22) at the real resonant frequency $\omega_m = Re\{\tilde{\omega}_m\}$ and then substituted into the phenomenological Fano formula:

$$\sigma_{Fano} = \sigma_0 \left[\frac{q^2 - 1 + 2q\Omega_m}{(\Omega_m^2 + 1)(q^2 + 1)} \right] + \sigma_{bg}, \quad \text{(S24)}$$

where $\sigma_0 = \sigma_m$, $q = q_m$. Here individual QNM responses are studied, thus $\sigma_{bg} = 0$. All RFA calculations were performed using the COMSOL eigenfrequency solver together with MAN or Python scripts (see links in Section III below).

Figure S2(a) shows the comparison between exact and RFA QNM responses for some low-order Mie modes (up to quadrupoles). Figure S2(b) shows the root mean square error dependence on Q factor for line shapes of the dipole and quadrupole Mie-modes for four particles with permittivity $\varepsilon = 9$, 12, 14, 16.

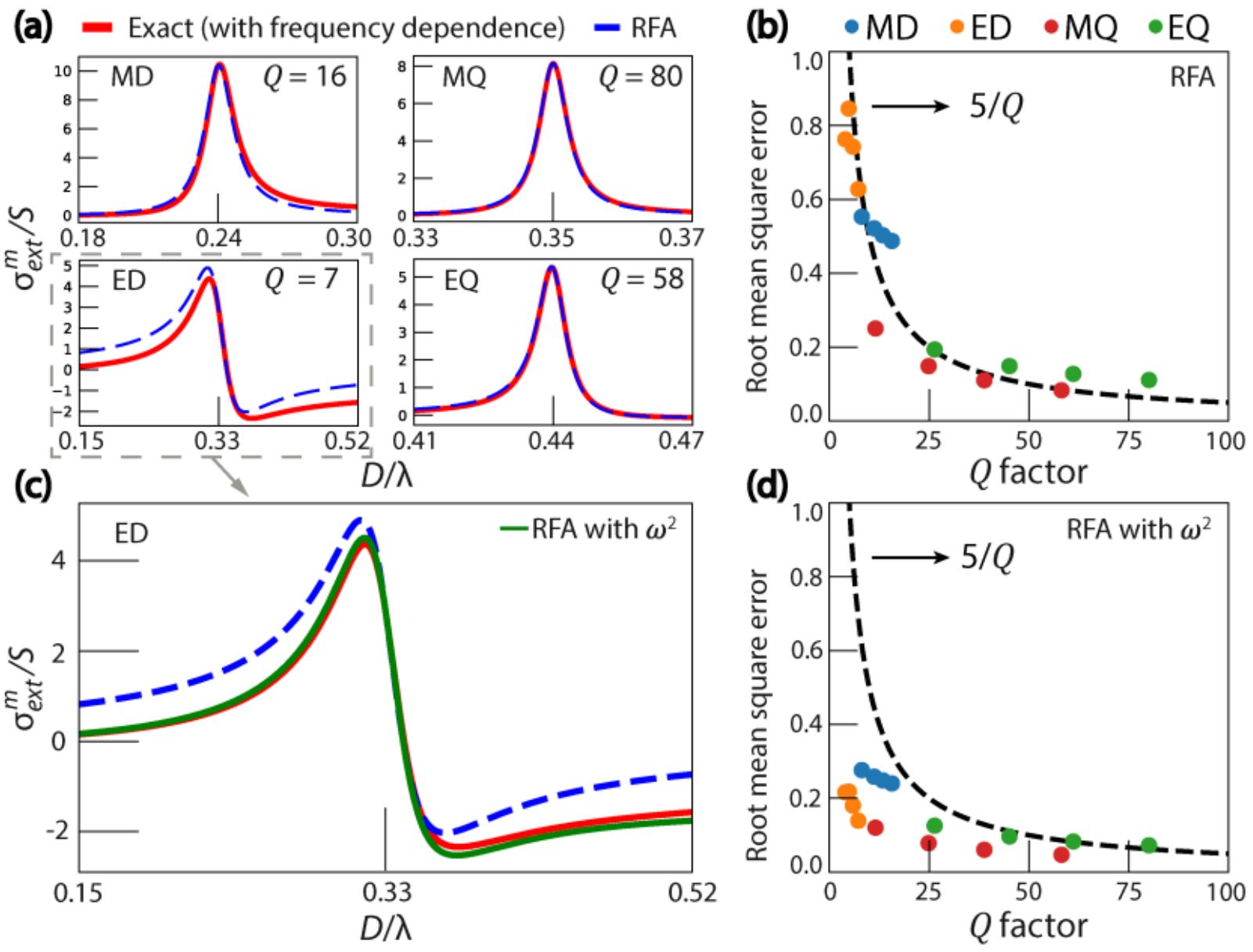

Fig. S2 – **(a)** Comparison between exact (red solid curves) and RFA (blue dashed curves) QNM responses for MD, ED, MQ and EQ for a spherical particle with $\varepsilon = 16$. **(b)** Root mean square error for the RFA dependence on the Q factor of modes for spheres with $\varepsilon = 9$, 12, 14, 16; the black dashed curve is a guide for eyes that depicts the hyperbolic function $5/Q$. **(c)** Comparison of the exact QNM response for ED (red solid curve), the RFA response (blue dashed curve) and the RFA response that account the ω^2 −dependence of the intensity term σ_m (green solid curve). **(d)** Root mean square error dependence on the Q factor for RFA responses that account ω^2 −dependence of the intensity term σ_m in Eq. (S22). In (a) and (c) extinction spectra are normalized on particle cross section $S = \pi R^2$.

In this case, the accuracy of RFA is excellent for modes with $Q > 50$. However, it seems that for modes with low Q, e.g. the magnetic dipole (MD, $Q = 16$) and especially the electric dipole (ED, $Q = 7$), the RFA cannot be applied if a precise prediction of the Fano line shape is required.

On the other hand, we can consider a modified RFA approach that accounts for the impact of the ω^2 −dependence within the intensity σ_m in Eq. (S22), while all other terms are evaluated at the real resonant frequency (Figs. S2(c, d)):

$$\sigma_{Fano} = \omega^2 \sigma_0 \left[\frac{q^2 - 1 + 2q\Omega_m}{(\Omega_m^2 + 1)(q^2 + 1)} \right] + \sigma_{bg}, \tag{S25}$$

where $\sigma_0 = |\xi_m(\omega_m)|/(2\gamma_m P_0)$, $q = q_m$ and $\sigma_{bg} = 0$. Comparing Figs. S2(b) and (d) reveals that the root mean square error decreases by approximately two to four times for resonances with $Q < 25$. The line shape (green curve) for ED resonance (Fig. S2(c)) becomes much closer to the exact solution (red curve), than the standard RFA (blue curve). All other imperfections occur due to neglecting the frequency dependence of the term ξ_m. Thus, for non-dispersive materials the RFA provides accurate results for resonances with $Q \geq$ 5-10, which can be considered as an empirical condition for applicability. Indeed, better accuracy of the line shapes can be obtained by taking into account the ω^2 −factor in σ_m.

The next point is a material dispersion, which can make an impact on q_m and σ_m. For the demonstration, we consider plasmonic dolmen made of Ag (silver) (Fig. S3). The parameters of the structure are the same as in [6] and COMSOL model to compute and normalize the QNMs can be found in the MAN package examples [7]. Permittivity of Ag can be described by Drude model $\varepsilon_{Ag} = \varepsilon_{\infty,Ag} - \frac{\omega_p^2}{\omega(\omega + i\gamma)}$ with parameters for: $\varepsilon_{\infty,Ag} = 1$, $\omega_p = 1.3659 \cdot 10^{16}$ rad/s, $\gamma = 0.0023 \cdot \omega_p$ (Fig. S3(b)). The extinction cross section normalized to the cross section of the smallest Ag cuboid $S = 30$ nm $\cdot$ 100 nm. The Q factors for modes 1,2 and 3 equals: Q_1 = 19.8, Q_2 = 22.6, Q_3 = 8.8. For simplicity, we consider in-plane rotations of the resonator, relative to incident wave (Fig. S3(a)).

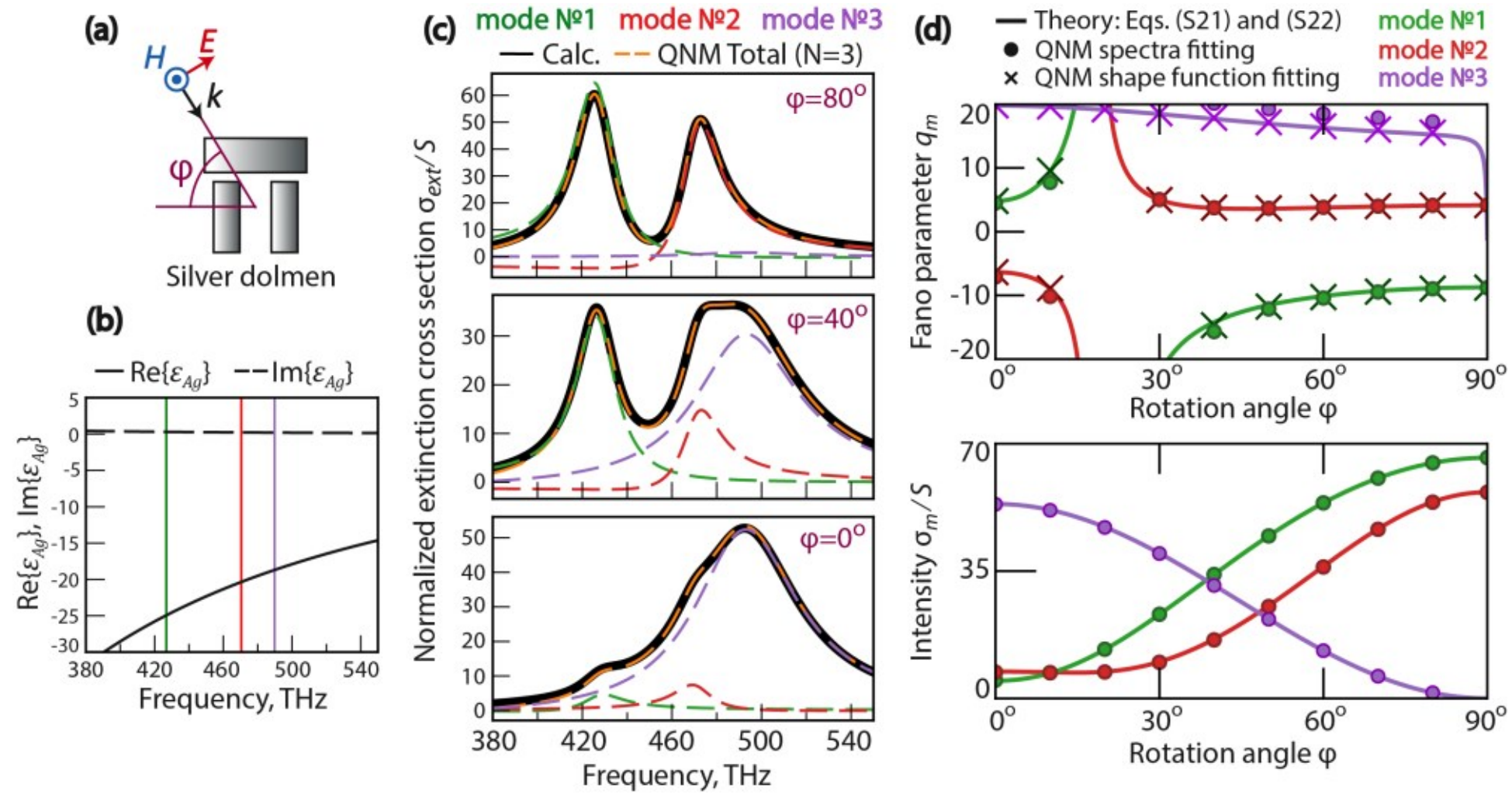

Fig. S3 – **(a)** Excitation geometry. **(b)** Frequency dependence of the permittivity of Ag, three colored curves marks frequencies of mode № 1,2, and 3. **(c)** Total normalized extinction cross section calculated with COMSOL frequency-domain solver (black curves) and QNM decomposition for three modes with Eq. (S20) (dashed color curves). **(d)** The Fano parameter and intensity dependence on the rotation angle φ, present theory (Eqs. (S21) and (S22), RFA) and fit (Eq. (S25), $\sigma_{bg} = 0$) for modes №1, 2, and 3. $Q_1 = 19.8, Q_2 = 22.6, Q_3 = 8.8$.

We again compare the exact QNM response (Eq. (S20)) and the corresponding Fano fit (Eq. (S24), $\sigma_{bg} = 0$) with the RFA calculation of σ_m and q_m (Eqs. (S21-22)). Fig. S3(d) demonstrates that the RFA is applicable for a dispersive case. For intensities of all three modes, we observe good agreement between Fano fitting and RFA. For the Fano parameter, a minor divergence arises when we compare the extinction spectra fit (circles in Fig. S3(d)) with the RFA calculations. This divergence occurs mostly due to the ω^2 −dependence of the intensity term σ_m. If one calculates the shape functions of QNM responses $\left[\frac{q_m^2-1+2q_m\Omega_m}{(\Omega_m^2+1)(q_m^2+1)}\right]$ with Eq. (S20) and fit these curves using Eq. (S24) with $\sigma_0 = 1$, then the perfect agreement can be achieved (crosses in Fig. S3(d)). For the shape functions, there is no impact of the intensity term, but the Fano parameter remains the same.

Conclusion. The divergence between line shapes of the exact solution and the RFA becomes valuable when Q factors of resonances are less than 50. The strongest impact on the error occurs due to the ω^2 −contribution inside the σ_m term, which distorts the response line shape; a weaker impact has a frequency dependence of the term ξ_m. The considered root mean square error depicts the total difference between exact and approximated line shapes. One can observe that in the Figs. S2-S3, terms σ_m and q_m are still well predicted by RFA for low-Q modes. Note that the RFA provides *exact* results at the resonant frequency. Therefore, the RFA can be used to obtain σ_m and q_m even for low-Q resonances. As for spectral line reconstruction, the empirical condition for applicability of the RFA is $Q \geq 5$ for non-dispersive particles. For a case with material dispersion, the condition for applicability may be different, at least for the Ag-dolmen the RFA is still able to obtain accurate results for modes with $Q \sim 10$. Notable, that the Eqs. (S20) and (S22) are derived with great generality, therefore they guarantee that Eq. (S25) will always provide better fit than Eq. (S24) for a resonant line in an extinction spectrum. And the last important conclusion is that the equation for Fano line shape is general and should not be modified even for low-Q states in light scattering problems, because Eq. (S20) without the RFA is a Fano line shape and always provides exact results.

II.2 Frequency sampling and computational cost analysis

Choosing the optimal frequency resolution based on the tradeoff between adequate spectral resolution and computational time is a non-trivial task. This choice depends heavily on the specific case and the purpose under consideration. In this section, we provide a brief comparison of the performance of RFA and a standard frequency-domain solver in terms of computation time to draw simple conclusions, which may simplify this choice. This comparison is performed for the cylindrical resonator used in Section 2 of the main text. We note that we used only P-Cores (high-performance) of our processor Intel Core i7-13700K (64 GB of DDR4 RAM), to prevent accidental switch between E-cores (energy effective) and P-cores, which has impact on computational time.

Test of computational performance. To clarify the comparison of computational costs, we present in Fig. S4(a) extinction spectra calculated with the standard COMSOL solver using five different numbers of frequency points (fp), ranging from 101 to 2001. Figure S4(b) shows an enlarged wavelength range (800–880 nm) containing two high-Q resonances (VII with $Q = 224$ and VIII with $Q = 109$); the corresponding number of fp within this interval is indicated in the figure. The computational times for the five cases are summarized in Fig. S4(c), demonstrating an

almost linear dependence on the number of fp for the standard method in this case: the average computational speed is defined as $2001/(47\,\text{min}\,03\,\text{sec}) \sim 42.5$ fp/min. From Fig. S4(b), it is clear that 1501 fp are almost sufficient to adequately resolve the full spectrum and the two resonances. Therefore, if one is specifically interested in these two features in the enlarged wavelength range, the computational time of the standard COMSOL simulation can be reduced accordingly to $86/(42.5\ \text{fp/min}) \sim 2$ min. However, the main difficulty is that, a priori, one does not know which frequency resolution will be sufficient or how many resonant features are present in the selected spectral range.

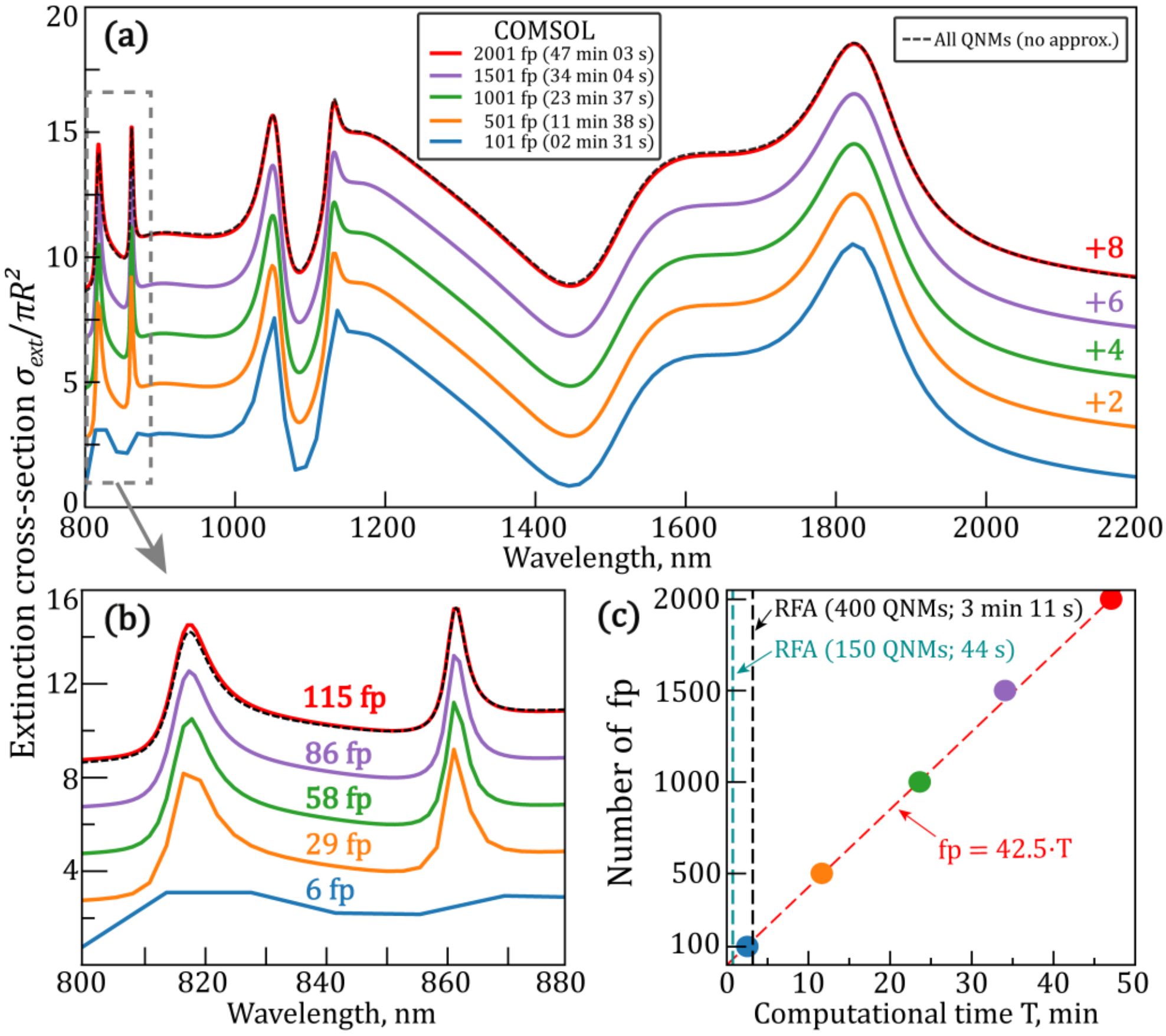

Fig. S4 – Comparison of computational time and spectral representation quality depending on the number of frequency points (fp). **(a)** Five extinction spectra of the cylindrical particle (described in Section 2 of the main text) calculated using different numbers of fp, ranging from 101 to 2001. Colored numbers on the right indicate the vertical offsets applied for clarity. **(b)** Enlarged wavelength range from 800 to 880 nm containing two high-Q resonances: VII (Q = 224) and VIII (Q = 109). The corresponding number of fp within this range is indicated. **(c)** Computational time for the five spectra calculated with the standard COMSOL solver, compared with RFA calculations using different numbers of QNMs: cyan dashed line corresponds to 150 QNMs and black dashed line to 400 QNMs. The red dashed line represents a linear dependence of the computational speed (fp/min) for the standard solver, in this case defined as $N/T = 2001/(47\ \text{min}\ 3\ \text{s}) \sim 42.5$ fp/min. Black dashed curves in (a) and (b) represent QNM reconstruction without RFA.

One of the key advantages of the QNM and RFA formalism is its practical independence from the number of frequency sampling points. The dominant computational cost arises from solving the eigenvalue problem. Once the eigenmodes are obtained, the spectral response is reconstructed

semi-analytically using Eqs. (S20–22). All integrals entering the coefficients ξ_m are evaluated using Gaussian quadrature, i.e., by reducing the integration to a weighted summation, which enables very fast evaluation [7]. For the RFA approach, these integrals calculated only once at $\omega_m = Re\{\tilde{\omega}_m\}$. As a consequence, RFA calculations performed with different frequency resolutions (101, 501, 1001, 1501, 2001 or even 20001 points) require essentially the same computational time (3 min 11 s when 400 QNMs are included, black dashed line in Fig. S4(c)). For demonstration, all RFA examples in this section we used 20001 fp.

Computational time of RFA method can also be optimized by reducing the number of QNMs considered. For example, using 150 QNMs decreases the total computation time to 44 s, with virtually no degradation in reconstruction quality for the present case (Fig. S5(a, b)). However, further reduction may result in the loss of certain resonant features, like in the case of 50 QNMs (orange curve in Fig. S5(a, b)). Here again, the challenge is that one does not know in advance how many QNMs must be included. Moreover, perfect reconstruction cannot generally be achieved within the RFA framework, due to its approximate nature.

Overall, a direct comparison between the two approaches is not straightforward. Both methods can be optimized in terms of computational time, and in both cases the optimal strategy strongly depends on the specific problem and may compromise the representation of spectral features. While RFA does not yield an exact reconstruction, it provides accurate approximation together with physical insight. Therefore, RFA becomes useful for rapid generation of highly resolved wide range spectra, including for different geometries or illumination conditions, where the perfect reconstruction is not required.

All these conclusions remain valid for more complex cases, such as the plasmonic example presented in Section 6 of the manuscript. In that case, the 100 QNMs + RFA calculations required approximately ~6 min, whereas the standard frequency-domain solver required 24 minutes for 101 fp (or ~5 min for 21 fp with the average speed 101/(24 min)~4 fp/min).

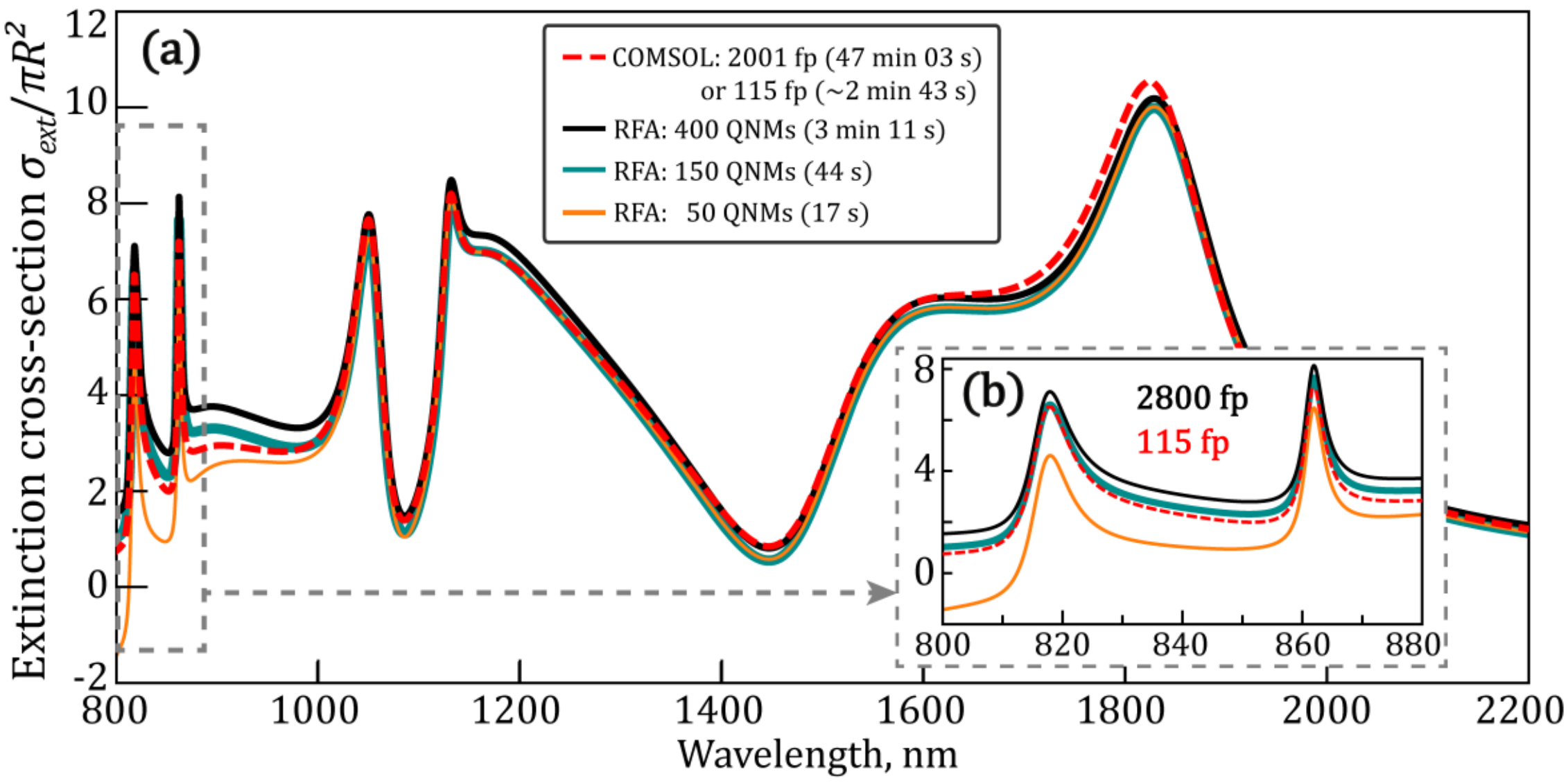

Fig. S5 – **(a)** Comparison of the quality of spectra reconstruction with RFA for different number of QNMs taken into account: orange curve 50 QNMs, cyan curve 150 QNMs, and black curve 400 QNMs. Insert **(b)**: enlarged wavelength range from 800 to 880 nm containing two high-Q resonances: VII ($Q = 224$) and VIII ($Q = 109$). The corresponding fp within this range is indicated. For total spectra from 800 to 2200 nm for all RFA calculations 20001 fp were used.

III. Performance of the theory: Demonstration for a few different cases

In this section, we provide additional details of calculations and demonstrate the performance of the present theory (Eqs. (S21) and (S22)) for several scenarios, including various resonator shapes and materials and the presence of a substrate:

- First, we provide technical details about calculations of the exceptional point in ring with a groove defect.
- Second, we examine the dielectric split-ring resonator used in the experiment, comparing the predictions from Eqs. (S21) and (S22) with numerical results rather than with the experimental results reported in the main text.
- Third, we analyze a dielectric cylindrical resonator that exhibits a quasi-bound state in the continuum. This example is taken from [8].
- Fourth, we consider a dielectric particle placed in a hollow metallic waveguide. This example shows that the present theory is applicable to the transmittance spectra of waveguides. The example is inspired by work [9].
- Fifth, we explore the practical case of resonators on substrates, using the same dielectric split-ring resonator as in the second example.
- Sixth, a plasmonic nanoresonator on a substrate is considered as an example of an optical sensor, the characteristics of which were optimized using the resonant frequency approximation (RFA) method. This example inspired by works [10],[11].
- Lastly, we consider the most challenging case of a dispersive perovskite nanoparticle in a layered substrate.

All our calculations utilized the COMSOL eigenfrequency solver together with the freeware MAN (based on MATLAB) or custom Python scripts. Computations were performed on a standard personal computer with the Intel Core i7-13700K processor and 64 GB of DDR4 RAM.

The advanced implementation of present theory is available in the MAN version 9.0 or later. The Fano parameters can be computed with the new function "getAmplitude_and_qFano_RFA.m". Modal contributions to extinction spectra are computed with "getExtinctionFano.m", which implements the classical Fano formula in Eq. (S20). These two functions are applicable within the reconstruction toolboxes to existing models in MAN, such as the nanoparticle-on-metal (NPoM) system and the plasmonic dolmen in free space [7] [https://zenodo.org/records/7400937].

Additionally, we developed Python implementation, which is designed to be accessible to beginners in QNM theory. These implementation includes Python codes, COMSOL model and instructions for calculation q_m and σ_m for cases of the split ring resonator in air and on a substrate, and for the dispersive plasmonic dolmen. This implementation is available at [https://github.com/Misha-Bochkarev/Fano-resonances.git].

III.1 Exceptional points in ring resonator with defect

In this section, we provide additional details for the exceptional point (EP) case discussed in the main text of our article. The results are shown in Fig. S6, where inserts (a-d) repeats the results, shown in the main text; (e-g) provides a more detailed comparison of spectral responses; (i) provides distributions of $\tilde{\mathbf{E}}_m$ (only the y-component with phase).

Figures S6(e-g) show that calculations with the standart "frequency domain" COMSOL solver (black solid curves) have a nice correspondence with QNM calculations (solid and dashed orange

curves), the latter utilize another COMSOL solver - the "eigenfrequency" solver. The slight differences between the black and orange solid curves in Figs. S6(e-g) are caused by this difference in solvers. Note that for this calculations we used the same mesh for both solvers.

Figure S6(i) presents the spatial distribution of the y-component of the QNMs electric fields. We focus of the y-component because the illumination condition is given by $\mathbf{E}_b = (0, \mathrm{E}_y, 0)^{\mathrm{T}}$ in our simulations, such that only the y-components contribute to the ξ_m term (Eq. (S9)). As shown in Fig. S6(i), the fields are close to exhibit a phase shift of $i = e^{i\pi/2}$ near EP, indicating that they approximately satisfy the relation $\tilde{\mathbf{E}}_1 \approx i\tilde{\mathbf{E}}_2$. In a limiting case, this leads to the following relation between the ξ_m terms: $\xi_1 = (i)^2\xi_2$, and consequently to $\sigma_1 = \sigma_2$ and the phase relation $arg(\xi_1)/2 = arg(\xi_2)/2 + \pi/2$. From the latter we obtain, $q_2 = -\cot(arg(\xi_2)/2)$ and $q_1 = \mathrm{tg}(arg(\xi_2)/2)$, hence the direct connection between Fano parameters is $q_1 q_2 = -1$.

Notably, the sum of two Fano resonances in the form of Eq. (S20) vanishes when $\sigma_1 = \sigma_2$, $q_1 q_2 = -1$, and $\tilde{\omega}_1 = \tilde{\omega}_2$, for any chosen q_1. These conditions therefore correspond to total destructive interference in Fano representation. When the equalities hold approximately, strong suppression of the combined response is observed, as illustrated in Fig. S6(e-g).

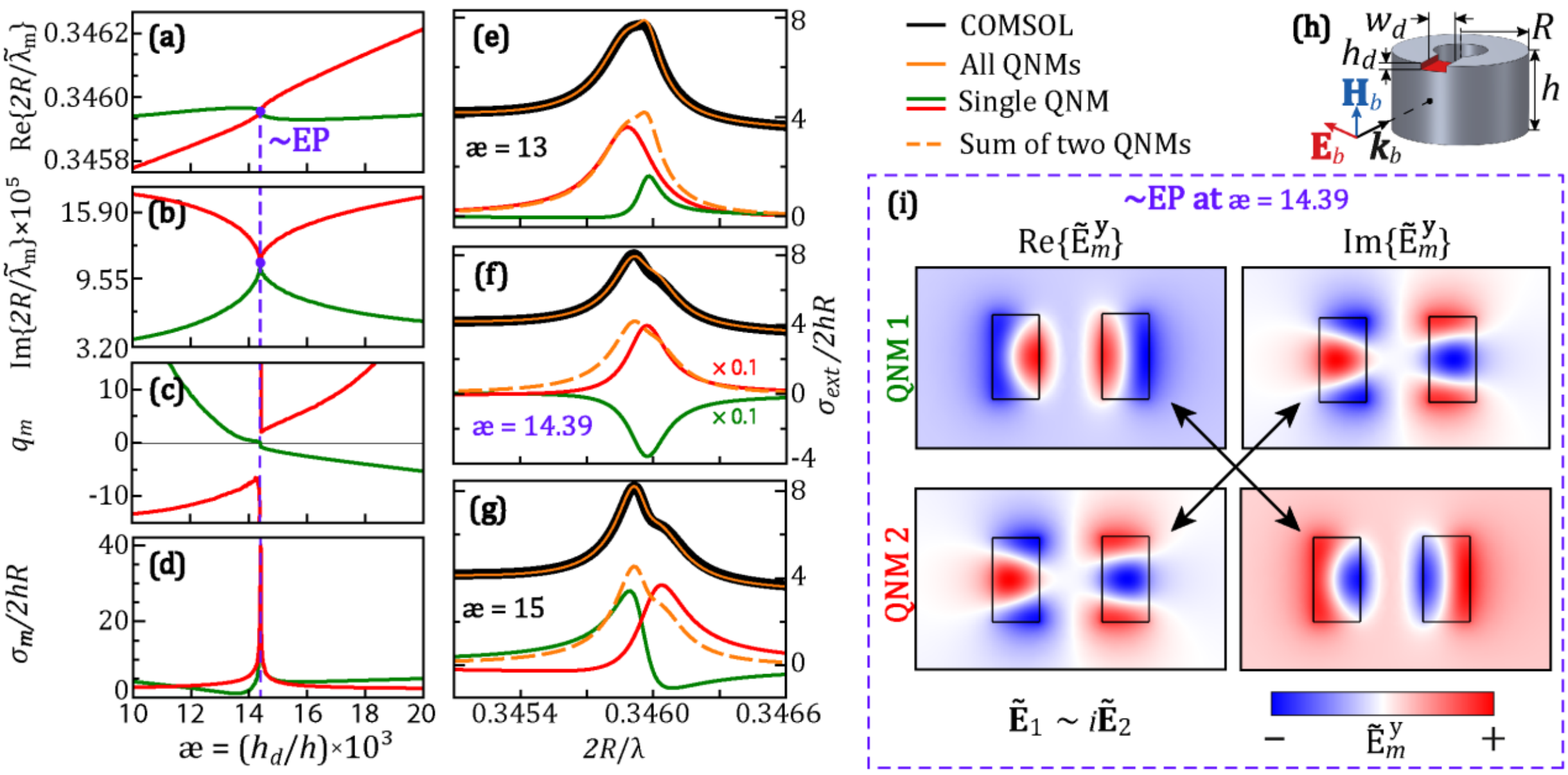

Fig. S6. – Behavior of spectral parameters near the exceptional point. **(a)-(b)** Real and imaginary parts of the resonance frequencies of the two coalescing QNMs as a function of the geometrical parameter æ = h_d/h×10³. **(c)-(d)** Evolution of the corresponding Fano parameters q_m and σ_m as æ varies. **(e)-(g)** Normalized extinction spectra for three different values of æ, æ = 13, 14.39 (very close to EP), and 15. The black curves represent COMSOL frequency-domain calculations; red and green curves correspond to the two QNMs; orange dashed curves depict their sum, while orange solid curves account for 200 QNMs. In (g), the red and green curves are scaled by 0.1 for better visualization. **(h)** Sketch of a ring resonator with a small groove air defect highlighted in red. Parameters used in the computation are: ε =43+0.003i for the ring, $\varepsilon_b = 1$, $r/R = 0.4$, $R/h = 0.894$, $R = 100$ nm, $w_d = r$, where r is the inner ring radius, and h_d is the variable. **(i)** Distribution of the y-component (real and imaginary parts) of the *normalized* QNMs fields.

The relations $\sigma_1 \approx \sigma_2$ and $q_1 q_2 \approx -1$ follows directly from the phase relation $\tilde{\mathbf{E}}_1 \approx i\tilde{\mathbf{E}}_2$, which we justify below using Perfectly Matched Layer (PML) norm [7]

$$N_m = \langle \Psi_m | \Psi_m \rangle = \int_{\Omega \cup \Omega_{\mathrm{PML}}} [\mathbf{E}_m \partial_\omega (\omega\varepsilon) \mathbf{E}_m - \mathbf{H}_m \partial_\omega (\omega\mu) \mathbf{H}_m] d^3\boldsymbol{r}. \quad \text{(S26)}$$

Exactly at an EP, Eq. (S26) yields $\langle \Psi_0 | \Psi_0 \rangle = 0$ due to the self-orthogonality of an EP eigenstate. To analyze the behavior of the PML norm in a neighborhood of an EP, we consider a system parameter $p = p_{\mathrm{EP}} + \delta p$, with $\delta p \to 0$. This parameter may correspond, for example, to the defect size.

There are two eigenfrequency branches $\omega_\pm$ with corresponding eigenvectors $|\Psi_\pm\rangle$, which can be expanded in the Jordan basis as

$$|\Psi_\pm\rangle = |\Psi_0\rangle \pm s|\Psi_1\rangle + \mathcal{O}(s^2), \quad \text{(S27)}$$

where $s \equiv s(\delta p)$ vanishes as $\delta p \to 0$. The sign of s reflects the two spectral branches and carries over to the eigenvectors. Equation (S27) can be viewed as a linear perturbative expansion in s for two QNMs near an EP.

Using Eq. (S27), we evaluate norms $N_\pm = \langle \Psi_\pm | \Psi_\pm \rangle$. Retaining only the dominant terms is s, we obtain $N_\pm \approx \pm s[\langle \Psi_0 | \Psi_1 \rangle + \langle \Psi_1 | \Psi_0 \rangle] \equiv \pm sC$, where $C \in \mathbb{C}$ is a constant. Consequently, $\sqrt{N_+ / N_-} \to i$, which leads to the phase relation between *normalized* fields

$$\tilde{\mathbf{E}}_+ \approx \frac{\mathbf{E}_{\mathrm{EP}}}{\sqrt{N_+}} \approx -i\frac{\mathbf{E}_{\mathrm{EP}}}{\sqrt{N_-}} \approx -i\tilde{\mathbf{E}}_-, \text{ or equivalently } \tilde{\mathbf{E}}_- \approx i\tilde{\mathbf{E}}_+. \quad \text{(S28)}$$

Therefore, near an EP, the phase relation $\tilde{\mathbf{E}}_1 \approx i\tilde{\mathbf{E}}_2$ arise from the normalization factor, when two nearly coalesced QNMs have norms $N_\pm$ differing by a phase of approximately π.

The same result can be obtained by considering a complex symmetric effective Hamiltonian

$$H = H^T = \begin{pmatrix} \widetilde{\omega}_1 & \kappa \\ \kappa & \widetilde{\omega}_2 \end{pmatrix}, \quad \text{(S29)}$$

where $\widetilde{\omega}_1$, $\widetilde{\omega}_2$, and κ are complex quantities. Such Hamiltonians are commonly used to describe open photonic systems (e.g., a resonator in free space). In the vicinity of the EP (but not exactly at the EP), the *normalized* eigenvectors acquire the same relative phase shift as derived above when the non-conjugated norm $N = \boldsymbol{v}^T\boldsymbol{v}$ is used, which is natural bilinear form for such systems.

Lastly, we provide an important technical note. Our numerical tests show that in studies of q_m, where a particle (or defect) shape changes occur, the mesh is crusially important in calculations of the PML norm [7]. The latter has a significant impact of the phase of $\tilde{\mathbf{E}}_m$, and hence on q_m. As a simple recommendation we suggest using the standart COMSOL mesh with the following settings: the "extremely fine" element size; "maximum mesh element size control parameter" equals $3f_0$, where f_0 is the frequency near QNMs of interest.

III.2 Resonances in single dielectric split-ring resonator

We observed a discrepancy between the theoretical prediction of q_m and σ_m and the experimental results in Fig. 4 from the main text. This inconsistency was explained by the spectral noise in the experiments. To justify the explanation, we calculated extinction spectra for the split-ring resonator (Fig. S7(a)) using the standard COMSOL frequency-domain solver (orange curves in Fig. S7(b)). Calculated results are in very good agreement with experimental spectra.

We then fit the calculated curves using Eq. (S24), where σ_{bg} is approximated by a second order polynomial in frequency and compare the fitted values with those computed with the present theory (Eqs. (S21) and (S22) which include the RFA). As shown in Figs. S7(c) and (d), the fit (orange dots) aligns almost perfectly with the theoretical results (solid curves). The slight discrepancy observed is due to the polynomial approximation of σ_{bg} used in the Fano fitting process.

This strong agreement confirms that the primary source of deviation between theoretical and experimental data in the main text is the spectral noise of the experiment, which reduces the accuracy of the Fano fitting.

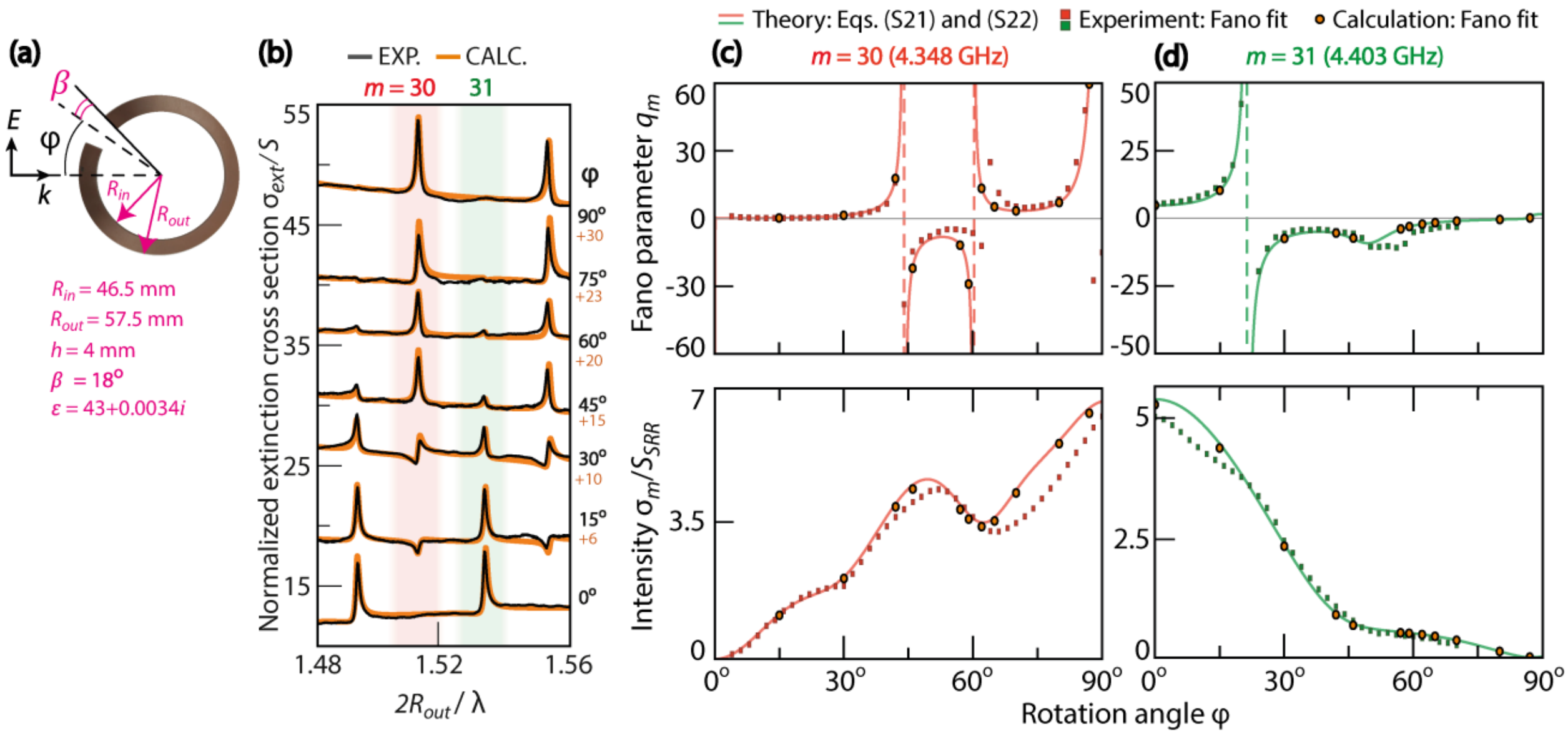

Fig. S7 – Comparison between theory and fitting of the numerical results for the QNM theory and FWS spectra. **(a)** Excitation geometry and parameters of the experimental sample. **(b)** Comparison between calculated (orange curves, COMSOL frequency-domain solver) and experimental spectra (black curves). **(c)** and **(d)** Comparison between the present theory and parameters from numerical (orange dots) and experimental (square dots) fit. Normalization constant in (b): $S = 2hR_{out}$

III.3 Scattering from a dielectric cylinder offering a bound-state in the continuum

Recently, a closed-from expression for the Fano parameter was derived in [8], which is the special case of Eq. (S23) for particles with spatial inversion symmetry, placed in homogeneous medium. The derivation in [8] is also based on QNM theory. Here, we reproduce results from [8] for a cylindrical particle with permittivity $\varepsilon = 80$, which offers a quasi-bound state in the continuum (qBIC) (insert in Fig. S8), showing that our method is stable upon changes in the resonator geometry.

Figure S8 shows the comparison between 1) fit parameters of the calculated spectra from [8] (blue curve); 2) the theory for q_m from [8] (circles); 3) The present theory for q_m calculated with Eq. (S21) with RFA (red dashed curve).

Figure S8 demonstrates that the fitting results align almost perfectly with those derived from Eq. (S21). In contrast, the theoretical predictions from [8] show a notable discrepancy when compared to the fitted values. This is caused by technical implementation of the method from [8], where $\tilde{\mathbf{E}}_m(\boldsymbol{r})$ was calculated approximately, while the present theory uses directly calculated $\tilde{\mathbf{E}}_m(\boldsymbol{r})$.

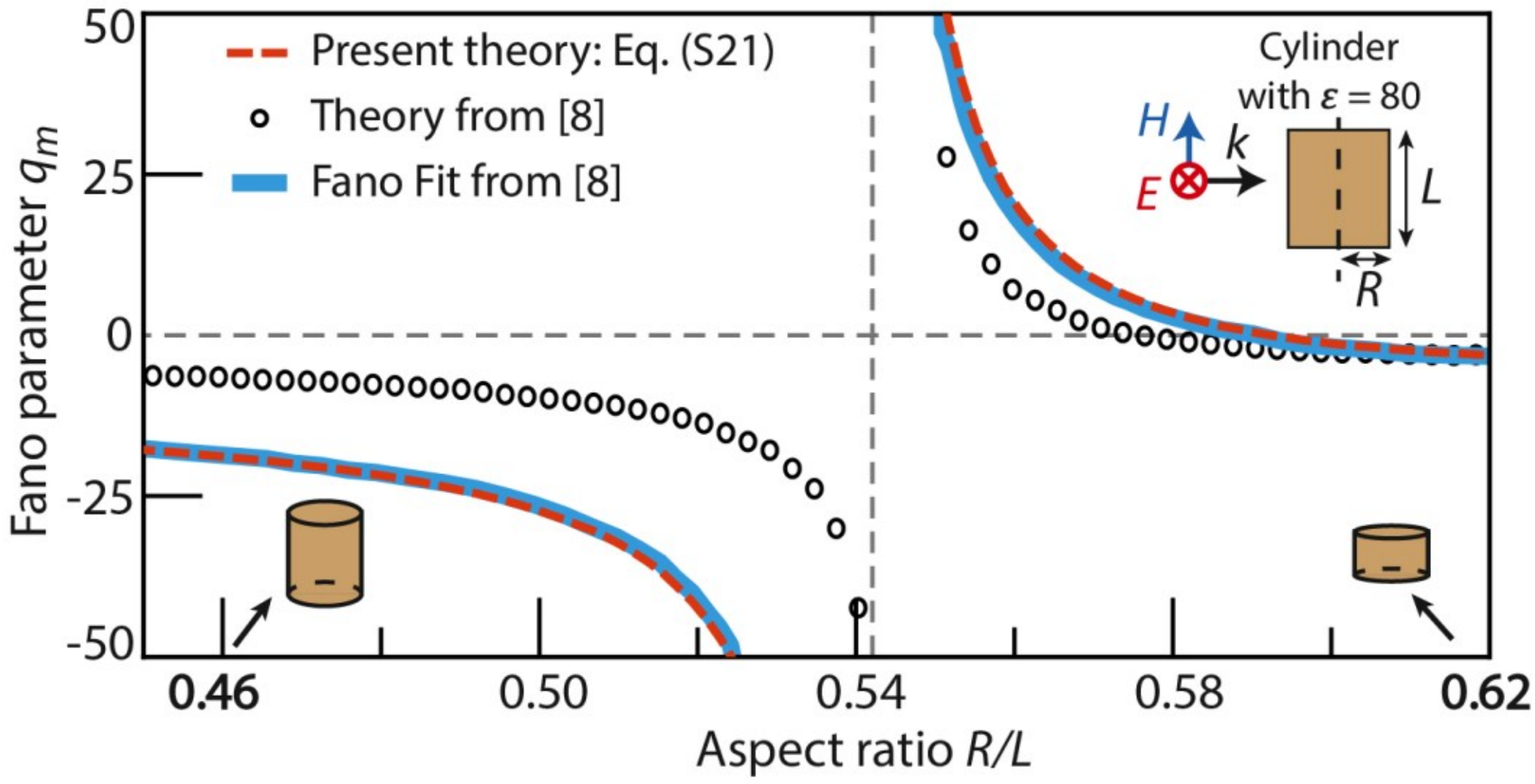

Fig. S8 – Dependence of the Fano parameter on the aspect ratio R/L for the TM_{111} mode. The blue curve and circles correspond to results from [8]; red dashed curve calculated by Eq. (S21) with the RFA. Grey dashed lines are guides for eyes. Insert (top right): sketch of particle and excitation setup.

The expression for the Fano parameter in [8] can be derived from Eqs. (S9) and (S23) under assumptions of (1) $\mathbf{E}_b$ is linearly polarized and (2) the spatial inversion symmetry of a corresponding QNM field component: $\tilde{\mathrm{E}}_{m,i}(-\boldsymbol{r}) = (-1)^p \tilde{\mathrm{E}}_{m,i}(\boldsymbol{r})$ with $i = x, y$ or z and the parity term $p = 0,1$. Thus, using a single overlap integral from Eq. (S9) and choosing for example $\mathbf{E}_b = \mathrm{E}_0 e^{ik_x x}\hat{\boldsymbol{y}}$ and $\tilde{\mathrm{E}}_{m,y}(-\boldsymbol{r}) = (-1)^p \tilde{\mathrm{E}}_{m,y}(\boldsymbol{r})$ one obtains

$$\kappa_m(\boldsymbol{k}_b = k_x\hat{\boldsymbol{x}}) \equiv \int_{V_r} \Delta\varepsilon \mathrm{E}_0 e^{ik_x x} \cdot \tilde{\mathrm{E}}_{m,y}(\boldsymbol{r}) d^3\boldsymbol{r} \underset{\boldsymbol{r}\to-\boldsymbol{r}}{\Longrightarrow} -\int_{V_r} \Delta\varepsilon \mathrm{E}_0 e^{ik_x(-x)} \cdot \tilde{\mathrm{E}}_{m,y}(-\boldsymbol{r}) d^3(-\boldsymbol{r})$$
$$= \int_{V_r} \Delta\varepsilon \mathrm{E}_0 e^{ik_x(-x)} (-1)^p \cdot \tilde{\mathrm{E}}_{m,y}(\boldsymbol{r}) d^3\boldsymbol{r}$$
$$= (-1)^p \int_{V_r} \Delta\varepsilon \mathrm{E}_0 e^{i(-k_x)x} \cdot \tilde{\mathrm{E}}_{m,y}(\boldsymbol{r}) d^3\boldsymbol{r} = (-1)^p \kappa_m(-\boldsymbol{k}_b). \quad \text{(S30)}$$

Therefore, under the assumptions, Eq. (S9) reduces to

$$\xi_m \equiv \left(\int_{V_{r'}} \Delta\varepsilon \tilde{\mathbf{E}}_m \cdot \mathbf{E}_b d^3\boldsymbol{r}'\right) \cdot \left(\int_{V_r} \Delta\varepsilon \tilde{\mathbf{E}}_m \cdot \mathbf{E}_b^* d^3\boldsymbol{r}\right)$$
$$= (-1)^p \kappa_m(-\boldsymbol{k}_b)\kappa_m(-\boldsymbol{k}_b) = (-1)^p \kappa_m(\boldsymbol{k}_b)\kappa_m(\boldsymbol{k}_b). \quad \text{(S31)}$$

Finally, substituting Eq. (S31) into the Eq. (S23) provides results from [8]

$$q_m = -\cot\left(\frac{\arg(\xi_m)}{2}\right) = -\cot\left(\frac{\arg\left((-1)^p \kappa_m^2\right)}{2}\right) = -\cot\left(\kappa_m + \frac{\pi p}{2}\right) \quad \text{(S32)}$$

III.4 Dielectric resonator in a hollow metallic waveguide

To show that RFA is applicable for particles placed in waveguides, we consider the ceramic plate-like particle with side $L = 20$ mm, thickness $H = 5$ mm, permittivity $\varepsilon = 80$, and losses $\tan\delta = 10^{-3}$. The particle is centered in the rectangular hollow metallic waveguide with dimensions $a = 34$ mm and $b = 72$ mm. For the background field $\mathbf{E}_b$ we consider TE_{10} mode, the host permittivity inside waveguide is $\varepsilon_b = 1$. The case is inspired by [9]. The results are shown in Fig. S9.

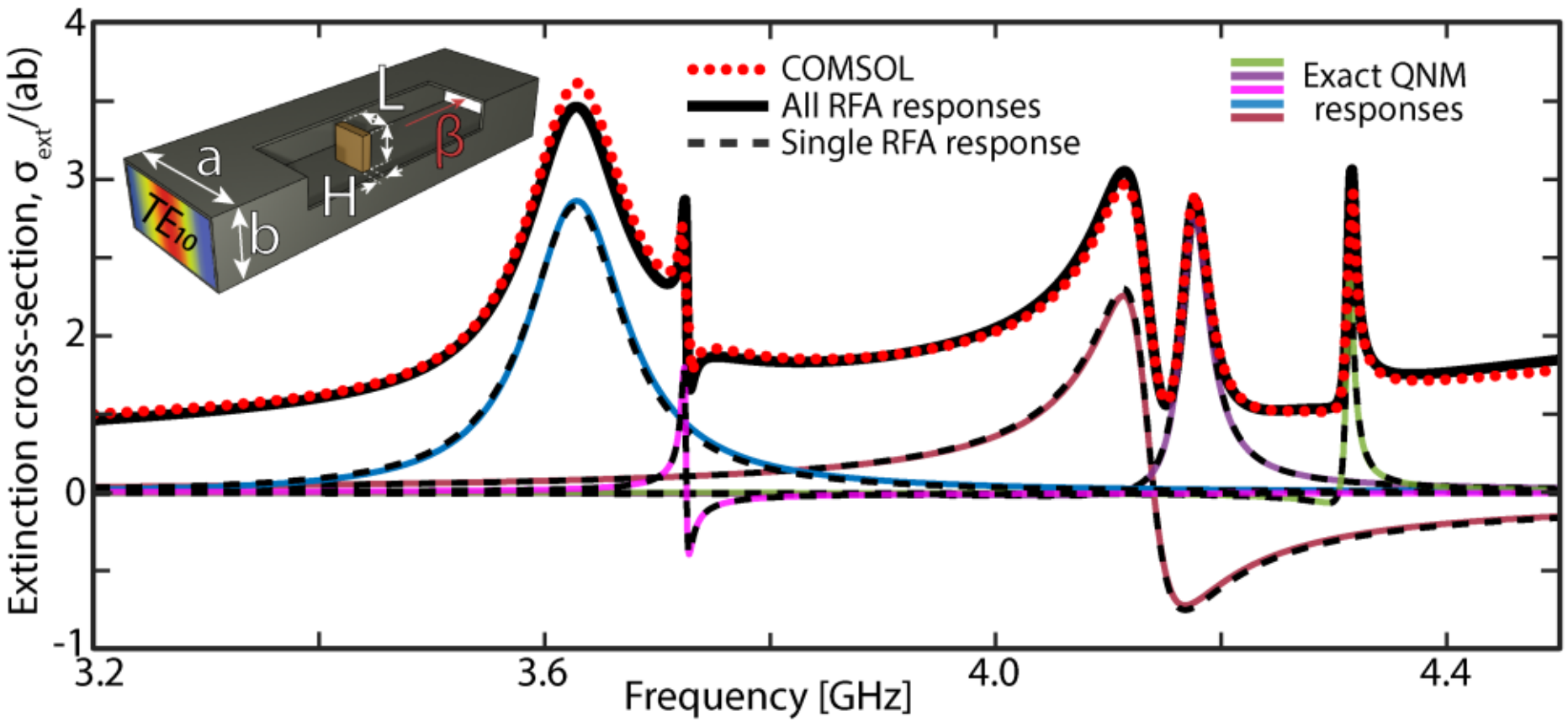

Fig. S9 – Performance of RFA in the reconstruction of extinction (or transmittance $Re\{S_{21}\} = 1 - \sigma_{ext}/(2ab)$, [9]) spectra for the particle in waveguide. Red dotted curve corresponds to the standard COMSOL frequency domain solver; black solid curve corresponds to RFA reconstruction. Other colored curves depict individual QNM responses, while dark dashed curves show the RFA results (analytical $\omega^2 -$ dependence from Eq. (S22) is preserved).

The strong agreement between RFA (black curve) and the standard 2-port frequency-domain COMSOL calculation (red dotted curve) in Fig. S9 confirms applicability of the present theory for studies of Fano resonances in waveguides. More details about this case, including experimental conformation of the present theory for q_m in the case of Fabty-Pérot BICs in such waveguides, can be found in [9].

III.5 Scattering from a dielectric split-ring resonator on a substrate

In QNM theory, the presence or absence of a substrate plays a critical role, as substrates introduce branch cuts in the complex frequency plane [2]. Physically, the presence of a substrate creates two distinct leakage pathways into the two separate half-spaces. QNM theory is inherently general and applies to resonators interacting with layered substrates. Consequently, it is crucial that the generalized Fano theory, as expressed in Eq. (S20), remains valid for resonators on substrates. This ensures it can accurately account for leakage not only in uniform open spaces but also in semi-infinite half-spaces.

We considered the dielectric split-ring resonator, identical to those used in the experiment, on the substrate with a refractive index $n = 1.75$ (Fig. S10). We first calculate the Fano parameter and intensity with the present theory (RFA, solid curves in Fig. S10(c) and (d)). Then we compared the theoretical results with those obtained numerically with COMSOL frequency-domain simulations (Fig. S10(b)). The latter were fitted to obtain the Fano parameters (dots in Fig. S10 (c) and (d)). The Fano fit is performed with Eq. (S24), where σ_{bg} was approximated by a second order polynomial of the frequency. We achieve a perfect agreement, matching the precision observed in the case without a substrate.

The extinction cross section (Fig. S10(b)) and intensity (Fig. S10(d)) are normalized by the incident power flow on the spilt-ring resonator with a surface $S_{SSR} = (\pi - \beta)(R_{out}^2 - R_{in}^2)$. Compared to previous cases, the background field $\mathbf{E}_b$ consists of the incident plane wave combined with the plane wave reflected by the substrate. Additionally, the QNMs are now calculated by incorporating the substrate into the simulation.

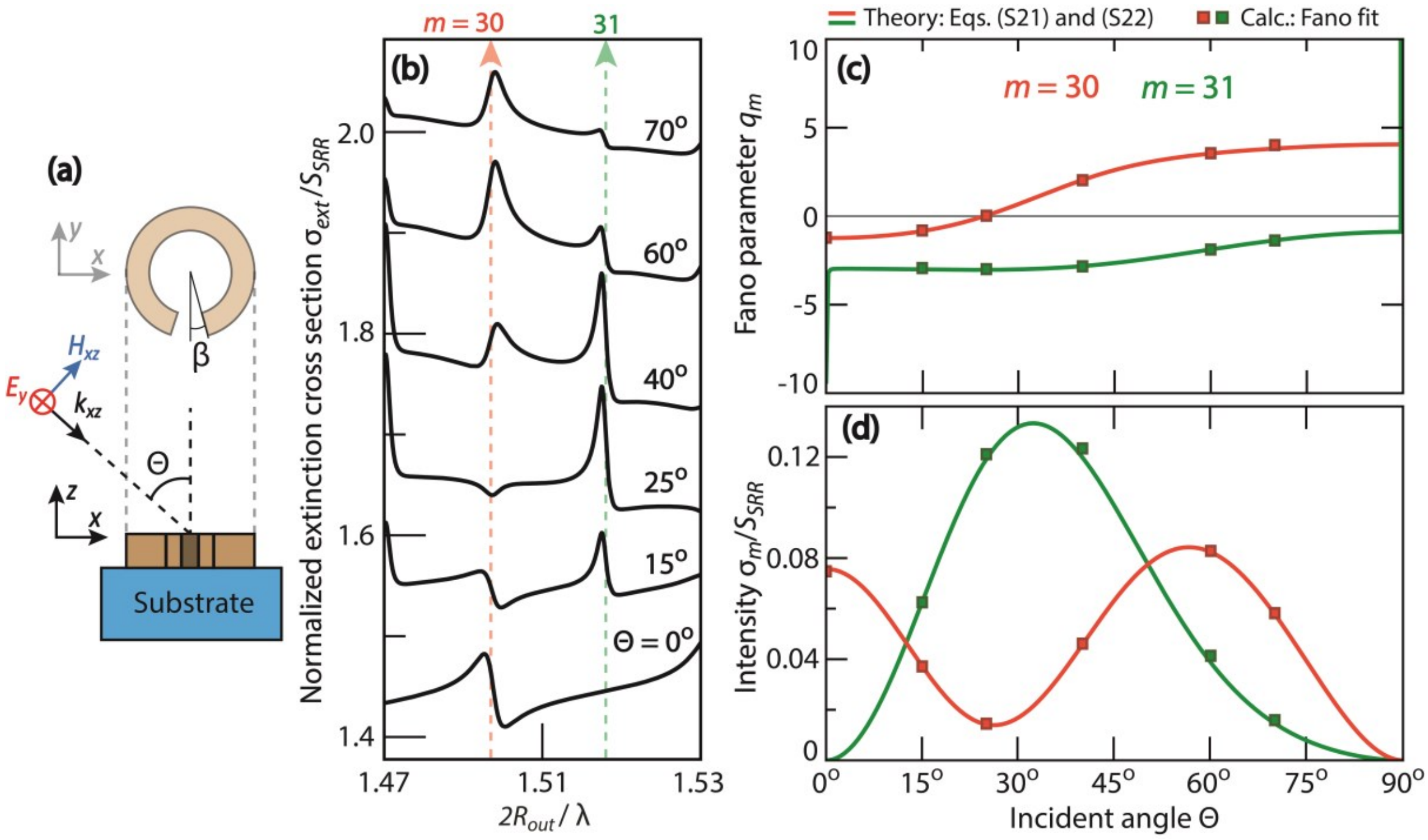

Fig. S10 – **(a)** Excitation setup with a split-ring on a substrate. **(b)** Normalized extinction cross section calculated with COMSOL frequency-domain solver for six different incident angles (shifted vertically for clarity). **(c)** and **(d)** The Fano parameter and intensity dependence on the incident angle Θ, the comparison between the present theory (RFA) and the Fano fit of calculated spectra.

III.6 Plasmonic nanoresonator on a substrate. Optical sensor optimization

In this section we demonstrate how the Limit of Detection (LoD) for optical sensors could be optimized by employing the present theory. According to [10], the optimal LoD is achieved when the figure of merit $Q\sigma_m$ is maximized, here intensity σ_m is usually obtained with Fano fitting. With the present theory, one can calculate exact value of σ_m with Eq. (S22) and provide fast sensor performance optimization. The time for finding the optimal structure can be reduced by orders of magnitude, as once the QNMs of the structure are evaluated, $Q\sigma_m$ can be readily computed. The optimal configuration is identified within a parameter space defined by the incident light angle and the substrate thickness. Results are shown in Fig. S11.

For the demonstration, we provide an example inspired by [11]: an oligomer composed of seven Ag-spherical particles with diameters $D = 100$ nm and distance between particles $d = 5$ nm, placed on gold substrate coated with a SiO_2 spacer ($n = 1.5$) and variable thickness T (insert in Fig. S11(b)). Numerical calculations are performed taking into account the dispersion of Ag and Au with Drude-Lorentz model. For the calculations, we employ the QNM auxiliary-field formulation [3] along with Eq. (S9). The computations are carried out with the "MAN" repository [7], utilizing MATLAB scripts to emulate COMSOL Multiphysics.

The structural optimization performed by varying of the spacer thickness T, which was changed from 100 nm to 300 nm. At each T in the range, the resonant frequency (Fig. S11(a)) and Q factor (Fig. S11(b)) were calculated, and simultaneously the intensity σ_m and the quantity $Q\sigma_m$ were calculated for different incident angles (Θ from 0° to 80°) of the excitation plane wave (Fig. S11(c)). Importantly, the optimal structure does not correspond to the one with the highest Q factor alone; instead, it is achieved with a design that balances both Q and σ_m [10].

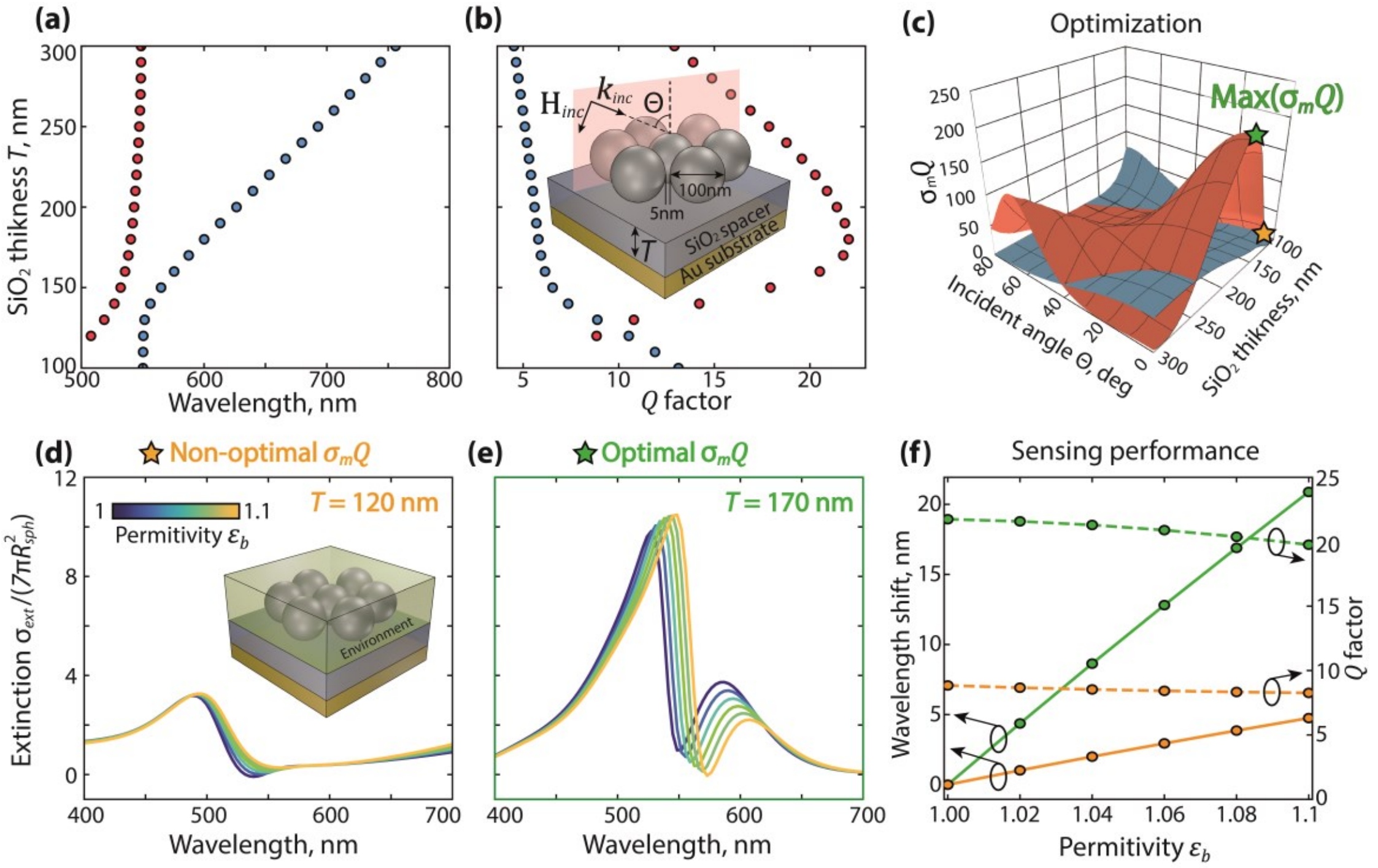

Fig. S11 – Scattering from an Ag-oligomer on a layered substrate. **(a)** Resonant wavelength and **(b)** Q factor dependence as a function of the spacer thickness T. Structure and excitation scheme are shown in the insert in (b). **(c)** $Q\sigma_m$ dependence on the thickness T and incident angle Θ for "red" and "blue" QNMs. Optimal LoD at $T = 170$ nm and non-optimal LoD at $T = 120$ nm are marked with green and orange stars, respectively. **(d)** and **(e)** Refractive index sensor performance: extinction spectra for the non-optimal (d) and optimal (e) thickness T upon changes in environment permittivity ε_b. **(f)** Resonant wavelength shift and Q factor dependence on ε_b for optimal (green curves) and non-optimal (orange curves) thickness T only for the "red" QNM from (a-c). For (d-f) the normal incidence is considered. Parameters of the structure: diameter of Ag-spheres $D = 100$ nm; separation distance between spheres $d = 5$ nm; spacer refractive index $n = 1.5$. Ag and Au material dispersions are considered in the computations.

In the chosen frequency range, there are two intense QNMs which were studied. As a result, for the "red" QNM, the optimal LoD is achieved at T = 170 nm at $\Theta = 0°$, for the "blue" QNM only moderate values are achieved in this range of parameters (Fig. S11(c)).

To test the sensing performance, we change the environment permittivity ε_b (inset in Fig. S11(d)) from 1 to 1.1 and compare extinctions spectra for two thicknesses $T = 120$ nm (non-optimal, Fig. S11(d)) and $T = 170$ nm (optimal, Fig. S11(e)). The oligomers are illuminated by plane waves with $\Theta = 0°$. For the optimal thickness, see Fig. S11(e), a 3× increase in intensity for the "Red" QNM is achieved. Simultaneously, the resonant wavelength shifts upon changes in ε_b is much stronger for the optimized structure (green curves in Fig. S11(f)), compared to non-optimal structure in Fig. S11(f)).

We emphasize that conventional numerical methods require recalculating high-resolution spectra for multiple parameter sets to achieve an accurate fit, making the Fano fitting process particularly challenging. In contrast, with the present theory, once the QNM is determined, σ_{ext}^{m}, σ_m, and q_m can be obtained analytically for any analytical $\mathbf{E}_b$. Notably, with COMSOL, the computational time for a single QNM calculation is equivalent to that required for computing extinction at a single frequency. This dramatically reduces the overall computational cost.

III.7 Dispersive perovskite nanoparticle on layered substrate

In this section, we examine the most challenging scenario: a dispersive nanoparticle possessing a material pole placed in a layered substrate. We assess the performance of the RFA approach for this scenario.

Figures S12(b,c) show a comparison between the extinction spectrum calculated with Eqs. (S20-S22) and an experimental dark-field scattering spectrum obtained by illuminating a $CsPbBr_3$ perovskite nanocuboid on an Al_2O_3/Ag substrate with unpolarized light (Fig. S12(a)). The experimental data are adapted from the supplementary materials in [12].

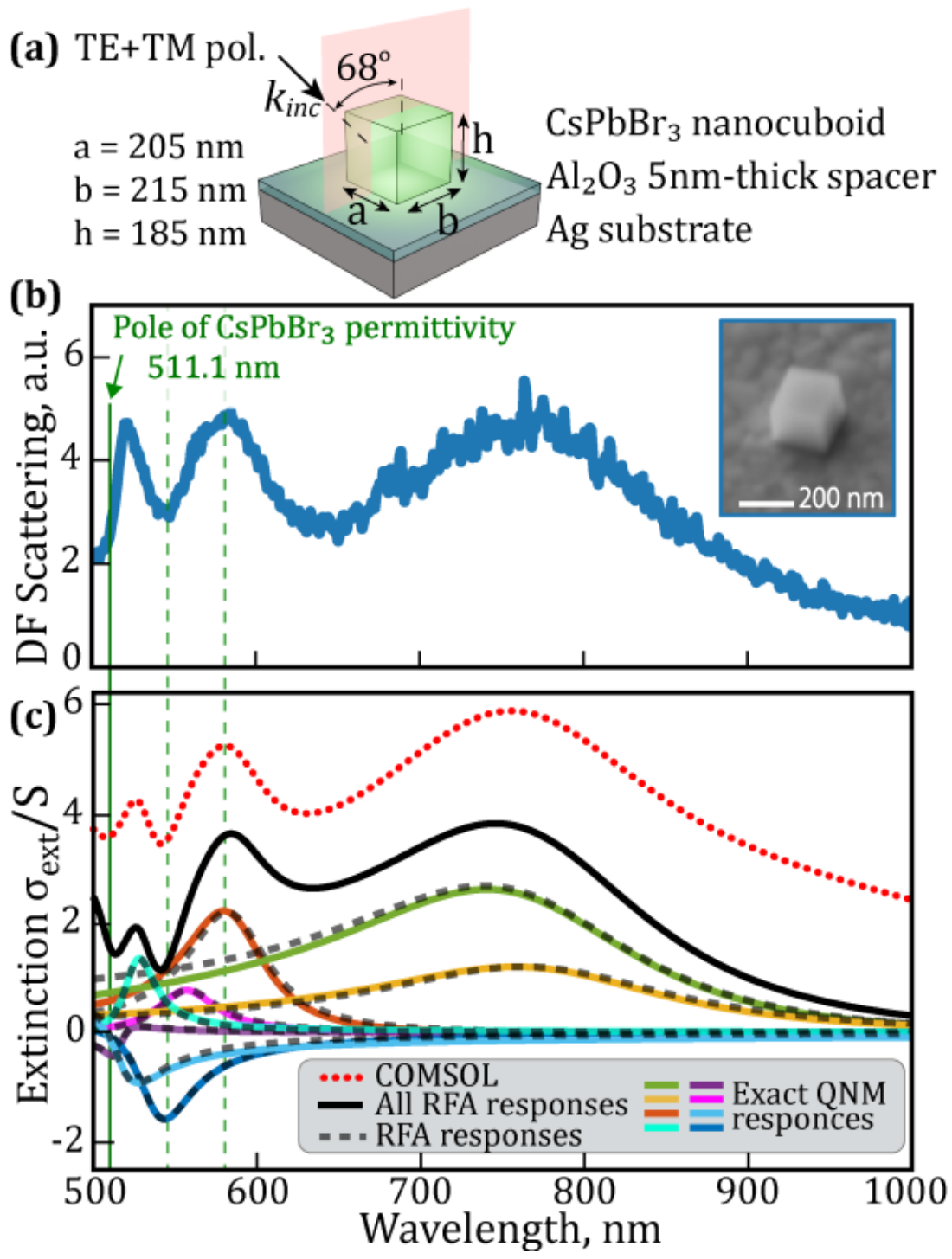

Fig. S12 – Comparison between QNM-calculated extinction spectrum and the experimental dark field (DF) scattering spectrum for a perovskite ($CsPbBr_3$) nanocuboid placed on a 5-nm Al_2O_3 spacer on the Ag substrate. **(a)** Sketch of the excitation scheme and parameters of the structure. **(b)** The experimental DF scattering spectrum at room temperature. Insert: scanning electron microscopy (SEM) image of the structure. **(c)** Extinction cross-section σ_{ext}/S, where $S = a \cdot b$, calculated with Eqs. (S20-S22). The total extinction σ_{ext} (black curve, 600 QNM contributions are accounted) corresponds to the linear sum of all individual RFA responses (dark dashed curves). The red curve represents spectrum, calculated with standard COMSOL frequency domain solver. Colored curves represent "exact" QNM responses σ_{ext}^{m}. In (b) the green solid line marks position of the perovskite permittivity pole at 511.1 nm; green dashed lines are guide for eyes. Note that $\mathbf{E}_b = \mathbf{E}_{inc} + \mathbf{E}_{ref}$, where $\mathbf{E}_{ref}$ is the reflected wave according to Fresnel equations. Experimental results in (b) are adapted from [12], under Creative Commons Attribution 4.0 International License.

Numerical calculations are performed for the nanocuboid parameters $a = 205$ nm, $b = 215$ nm, and $h = 185$ nm, averaging TE and TM-polarizations to effectively approximate unpolarized light, and taking into account the dispersion of $CsPbBr_3$ and Ag. The non-dispersive Al_2O_3 spacer is considered with refractive index $n = 1.7$ and thickness 5 nm. For the calculations, we employ the QNM auxiliary-field formulation [3] along with Eqs. (5-6) with RFA. The computations are carried out with the "MAN" repository [7], utilizing MATLAB scripts to emulate COMSOL Multiphysics, see details about materials properties and calculations below.

Figure S12 demonstrates a good agreement between the experimental spectrum in (b) and the theoretical predictions in (c). All key spectral features—including peak positions and asymmetries—are accurately reproduced by both standard COMSOL solver and RFA (red and black curves in (c), respectively). However, the RFA total response, as well as "exact" total QNM response (not shown), differs from the red curve by almost flat background intensity. This is caused by the presence of the permittivity pole (the accumulation point) of the perovskite, which generates an infinite set of QNMs in its vicinity and complicates the process of searching all QNMs, significantly contributing to the full spectrum. Despite the pole-problem, each individual "exact" QNM contribution (colored curves in Fig. S12(c)) is accurately recovered by the RFA (dark dashed curves), even near the material pole.

The presence of an accumulation point is one of the central challenges in modern QNM theory [14],[15] and, therefore constitutes the primary technical limitation of the RFA approach. A possible strategy to mitigate this issue is to employ advanced perturbation techniques [16]. For example, one may first compute the QNMs while neglecting the pole at 511 nm in the perovskite permittivity, and then subsequently introduce this pole as a perturbation that mixes the modal contributions in the spectrum. However, this is a technical and largely theoretical consideration that lies beyond the scope of the present work.

Description of materials properties. The dispersive properties of $CsPbBr_3$ are modeled using the N-pole Lorentz model [5]:

$$\varepsilon_{CsPbBr_3} = \varepsilon_{\infty,CsPbBr_3} + \sum_{j=1,2}\left[\frac{A_j e^{i\varphi_j}}{\omega-\omega_j} - \frac{A_j e^{-i\varphi_j}}{\omega+\omega_j^*}\right], \quad \text{(S33)}$$

where $\varepsilon_{\infty,CsPbBr_3}$, A_j, φ_j, ω_j – are fitting parameters, with index j corresponding to the pole of the permittivity. Using this model, the experimental permittivity data from [13] was reproduced with good quality, as shown in Fig. S13. The silver Ag-substrate was described by the Drude model:

$$\varepsilon_{Ag} = \varepsilon_{\infty,Ag} - \frac{\omega_p^2}{\omega(\omega+i\gamma)}, \quad \text{(S34)}$$

and the Al_2O_3 spacer is considered to be non-dispersive with refractive index $n_{Al_2O_3} = 1.7$. The material properties used for the calculations are listed in Table I.

Table I. Fitting parameters for ε_{CsPbBr_3} and ε_{Ag}.

Perovskite ε_{CsPbBr_3}	$\varepsilon_{\infty,CsPbBr_3} = 3$,<br>$A_1 = 0.1077 \cdot 10^{15}$ rad/s, $\varphi_1 = -2.8612$ rad, $\omega_1 = (3.6805-0.0484i) \cdot 10^{15}$ rad/s,<br>$A_2 = 3.1848 \cdot 10^{15}$ rad/s, $\varphi_2 = -3.1519$ rad, $\omega_2 = (5.0678-0.1196i) \cdot 10^{15}$ rad/s
Silver ε_{Ag}	$\varepsilon_{\infty,Ag} = 1$, $\omega_p = 1.3659 \cdot 10^{16}$ rad/s, $\gamma = 0.0023\omega_p$
Sapphire $\varepsilon_{Al_2O_3}$	$\varepsilon_{Al_2O_3} = 2.89$ ($n_{Al_2O_3} = 1.7$)

The QNM computation was performed by modifying the "QNMEig_NanolettSi.mph" model from the MAN package [7], which demonstrates the linearization of Maxwell's equations using auxiliary fields for systems exhibiting both types of dispersion (for ε_{Ag} and ε_{CsPbBr_3}).

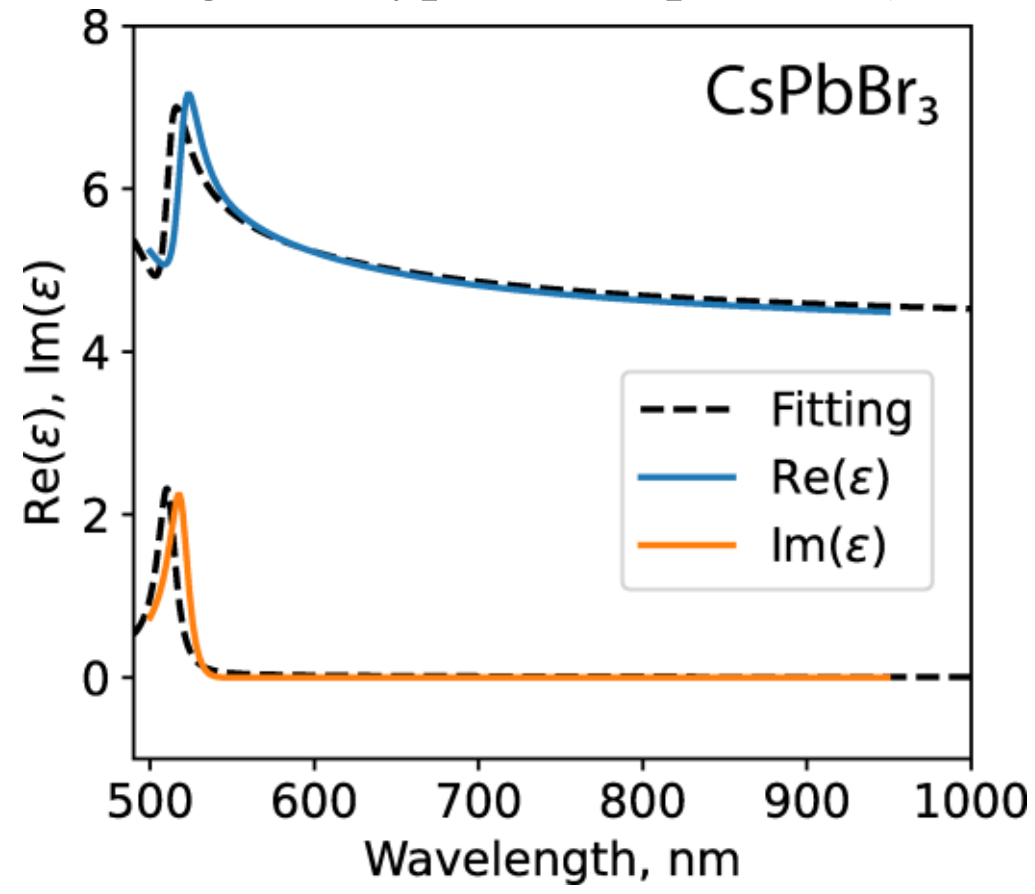

Fig. S13 – Experimental data (blue and orange curves) for the $CsPbBr_3$ permittivity [13] and corresponding fit (black curves) with the model from [5].

Computational issues. In total, 600 QNMs were included in the calculations; however, approximately only 20 of these modes provide significant contributions to the total QNM spectrum, while the remainder correspond to pole-associated QNMs of the perovskite. Notably, no PML-modes were found.

Because of the accumulation point, the computation of QNMs becomes comparable in runtime to a single standard COMSOL frequency-domain simulation and requires nearly 6 hours. Nevertheless, for parameter sweeps—where the incident field is varied but the resonant structure remains unchanged—the QNM approach regains a pronounced advantage. For example, calculation of the extinction map as a function of the incidence angle φ, shown in Fig. S14, consists of 90 spectra, which were generated in 28 minutes using the precomputed QNMs together with RFA (ω^2-dependence is preserved), whereas a single spectrum computed with the standard COMSOL frequency domain solver requires nearly 3 hours for 201 frequency points.

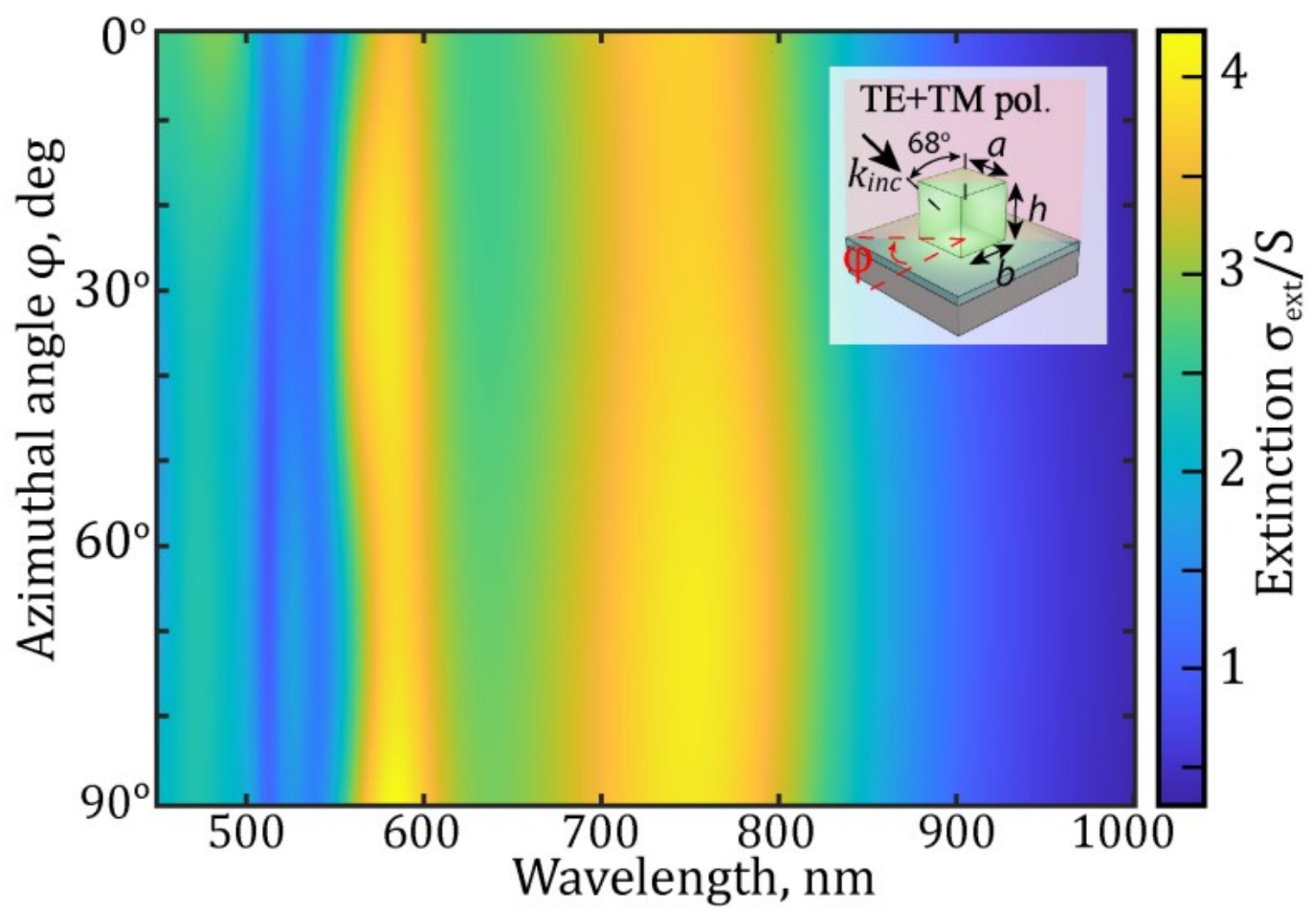

Fig. S14. Total RFA extinction spectrum of a perovskite nanocuboid on a substrate as a function of the azimuthal angle of incidence φ. The inset shows the direction of change of the angle φ.

IV. Experimental setup

Experimental measurements were conducted in the frequency range 1-8 GHz (wavelength range 30-300 mm) in an anechoic chamber (Fig. S15(a)), in which parasitic reflected radiation was absorbed. The setup for extinction cross section (σ_{ext}) measurements can be represented as a two-port system and described by a scattering matrix $\hat{S}$.

The sample was placed on a custom mount, which was made of transparent material in a chosen frequency range and located between two horn antennas (source and receiver) in the distance $L_S = L_R = 2$ m between each of them. The distance between antennas was $L = L_S + L_R = 4$ m.

The first antenna generated a plane wave, the second one received a transmittance signal, which corresponds to the S_{21} component of the scattering matrix and depicts the ratio between transmitted and incident electric field amplitudes. Horn antennas were connected to the "Rohde & Schwarz zvb20" vector analyzer. Such setup allows one to measure S_{21} components in present and absent of the sample, S_{21}^{sample} and S_{21}^{bg} correspondingly. After that, the extinction cross section can be obtained using the optical theorem [17]: $\sigma_{ext} = -\frac{4\pi L_R}{k_0} Im\left\{\frac{S_{21}^{sample}}{S_{21}^{bg}}\right\}$, where k_0 is the free-space wavenumber. To obtain angular dependencies of σ_{ext}, the custom mount was designed in such a way that it allows for rotation, relative to the direction of the incident plane, with the angle step of $\Delta\varphi = 1°$. This experimental setup represents the realization of a case when light scatters from a particle embedded in homogeneous media (air); the effects of the transparent mount-substrate are negligible, as confirmed by numerical simulations (see Fig. S7(b) in Section III.2 above). The photograph of the sample is shown in Fig. 4(a) in the main text.

Measurements of the near-field distribution of a magnetic field component H_z were provided in a similar manner, the only change is that a magnetic near-field probe (Fig. S15(b)) was implemented, instead of the receiving antenna. The probe provided point by point scanning of an area in the distance ~2 mm from the upper surface of the sample.

More details on the sample preparation can be found in [18].

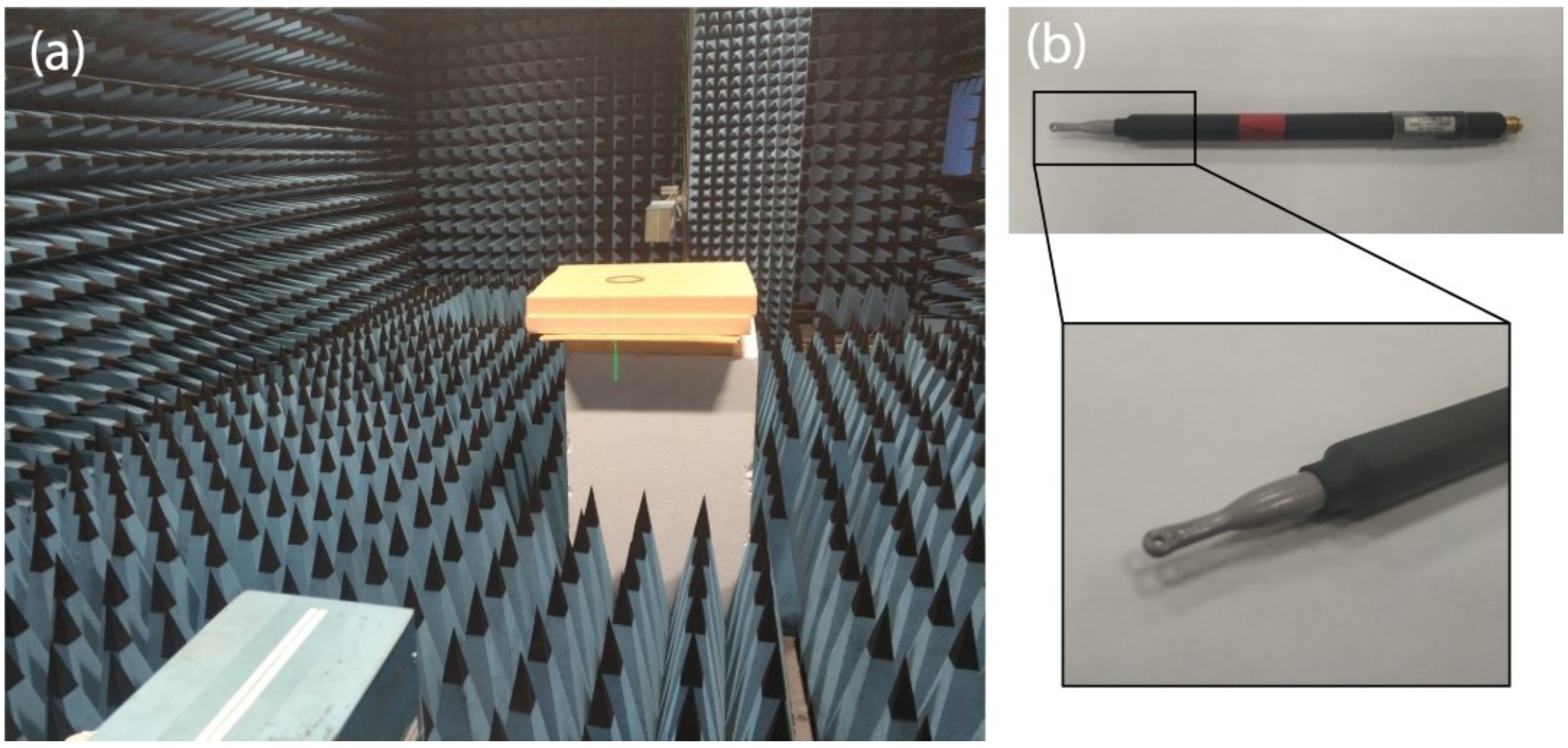

Fig. S15 – The experimental setup for microwave measurements. **(a)** Photograph of an anechoic chamber. The sample placed on transparent (in microwave range) mount on equal distance between source and receiver antennas is 2 meters, distance between antennas is 4 meters. **(b)** Magnetic probe for near field measurements.

References to supplemental document

[1] Q. Bai, M. Perrin, C. Sauvan, J.-P. Hugonin, and P. Lalanne, “Efficient and intuitive method for the analysis of light scattering by a resonant nanostructure,” *Opt. Express* **21**, 27371-27382 (2013).

[2] P. Lalanne, W. Yan, K. Vynck, C. Sauvan, and J.-P. Hugonin, “Light Interaction with Photonic and Plasmonic Resonances,” *Laser Photonics Rev*. **12**, 1700113 (2018).

[3] W. Yan, R. Faggiani, and P. Lalanne, “Rigorous modal analysis of plasmonic nanoresonators,” *Phys. Rev. B* **97**, 205422 (2018).

[4] A. Pick, P.R. Kaprálová-Žďánská, and N. Moiseyev, “Ab-initio theory of photoionization via resonances,” *J. Chem. Phys.* **150**, 204111 (2019).

[5] M. Garcia-Vergara, G. Demésy, and F. Zolla, “Extracting an accurate model for permittivity from experimental data: hunting complex poles from the real line,” *Opt. Lett.* **42**,1145-1148 (2017).

[6] T. Wu, A. Baron, P. Lalanne, and K. Vynck, “Intrinsic multipolar contents of nanoresonators for tailored scattering,” *Phys. Rev. A* **101**, 011803 (2020).

[7] T. Wu, D. Arrivault, W. Yan, and P. Lalanne, “Modal analysis of electromagnetic resonators: User guide for the MAN program,” *Comput. Phys. Commun.* **284**, 108627 (2023). Available at https://zenodo.org/records/7400937.

[8] A. Bogdanov, K. Koshelev, P. Kapitanova, M. Rybin, S. Gladyshev, Z. Sadrieva, K. Samusev, Y. Kivshar, and M. F. Limonov, “Bound states in the continuum and Fano resonances in the strong mode coupling regime,” *Adv. Photonics* **1**, 016001(2019).

[9] Z. Zhao, N. Solodovchenko, C. Sun, M. Song, E. Maslova, and A. Bogdanov, “Experimental study of Fabry-Perot BICs in a microwave waveguide,” *Appl. Phys. Lett.* **127**, 091701 (2025).

[10] D. Conteduca, G. S. Arruda, I. Barth, Y. Wang, T. F. Krauss, and E. R. Martins, “Beyond Q: The Importance of the Resonance Amplitude for Photonic Sensors,” *ACS Photonics* **9**, 1757-1763 (2022).

[11] N.A. Mirin, K. Bao, and P. Nordlander, “Fano Resonances in Plasmonic Nanoparticle Aggregates,” *J. Phys. Chem. A* **113**, 4028-4034 (2009).

[12] M.A. Masharin, D. Khmelevskaia, V.I. Kondratiev et al., “Polariton lasing in Mie-resonant perovskite nanocavity,” *Opto-Electronic Advances* **7**, 4, 230148 (2024).

[13] G. Ermolaev et al., “Giant and tunable excitonic optical anisotropy in single-crystal halide perovskites,” *Nano Lett.* **23**, 2570–2577 (2023).

[14] C. Sauvan, “Quasinormal modes expansions for nanoresonators made of absorbing dielectric materials: study of the role of static modes,” *Opt. Express* **29**, 6, 8268-8282 (2021).

[15] C. Sauvan, T. Wu, R. Zarouf, E. A. Muljarov, and P. Lalanne, “Normalization, orthogonality, and completeness of quasinormal modes of open systems: the case of electromagnetism,” *Opt. Express* **30**, 6846–85 (2022).

[16] E. Muljarov, W. Langbein, “Resonant-state expansion of dispersive open optical systems: Creating gold from sand,” *Phys. Rev. B.* **93**, 075417 (2016).

[17] C. Larsson and M. Gustafsson, “Wideband measurements of the forward RCS and the extinction cross section,” *ACES Journal* **28**, 8 (2013).

[18] N. Solodovchenko, M. Sidorenko, T. Seidov, I. Popov, E. Nenasheva, K. Samusev, M. Limonov, “Cascades of Fano resonances in light scattering by dielectric particles,” *Materials Today* **60**, 69-78 (2022).
